# SpoVID functions as a non‐competitive hub that connects the modules for assembly of the inner and outer spore coat layers in *Bacillus subtilis*

**DOI:** 10.1111/mmi.14116

**Published:** 2018-10-18

**Authors:** Filipa Nunes, Catarina Fernandes, Carolina Freitas, Eleonora Marini, Mónica Serrano, Charles P. Moran, Patrick Eichenberger, Adriano O. Henriques

**Affiliations:** ^1^ Microbial Development Laboratory, Instituto de Tecnologia Química e Biológica Universidade Nova de Lisboa Oeiras Portugal; ^2^ Department of Microbiology and Immunology Emory University School of Medicine Atlanta GA 30322 USA; ^3^ Department of Biology New York University New York NY 10003 USA; ^4^ Present address: Hovione, 2674‐506 Loures Portugal; ^5^ Present address: Department of Ecophysiology Max‐Planck Institute for Terrestrial Microbiology Karl‐von‐Frisch‐Str. 10 D‐35043 Marburg Germany

## Abstract

During sporulation in *Bacillus subtilis*, a group of mother cell‐specific proteins guides the assembly of the coat, a multiprotein structure that protects the spore and influences many of its environmental interactions. SafA and CotE behave as party hubs, governing assembly of the inner and outer coat layers. Targeting of coat proteins to the developing spore is followed by encasement. Encasement by SafA and CotE requires E, a region of 11 amino acids in the encasement protein SpoVID, with which CotE interacts directly. Here, we identified two single alanine substitutions in E that prevent binding of SafA, but not of CotE, to SpoVID, and block encasement. The substitutions result in the accumulation of SafA, CotE and their dependent proteins at the mother cell proximal spore pole, phenocopying a *spoVID *null mutant and suggesting that mislocalized SafA acts as an attractor for the rest of the coat. The requirement for E in SafA binding is bypassed by a peptide with the sequence of E provided in trans. We suggest that E allows binding of SafA to a second region in SpoVID, enabling CotE to interact with E and SpoVID to function as a non‐competitive hub during spore encasement.

## Introduction

All organisms face the challenge of assembling large numbers of proteins into functional cellular machines. Understanding the mechanisms underlying the assembly of supramolecular structures at specific locations during cell differentiation is a major goal in developmental biology. During endospore development by *Bacillus subtilis* and related organisms, a multiprotein, multilayered, structure called the coat is assembled around the surface of the developing endospore. This structure is formed by over 80 proteins and plays protective roles, while regulating germination and mediating interactions with the environment (Henriques and Moran, 2007; McKenney *et al*., 2013; Driks and Eichenberger, 2016; Setlow *et al*., 2017).

**Figure 1:**
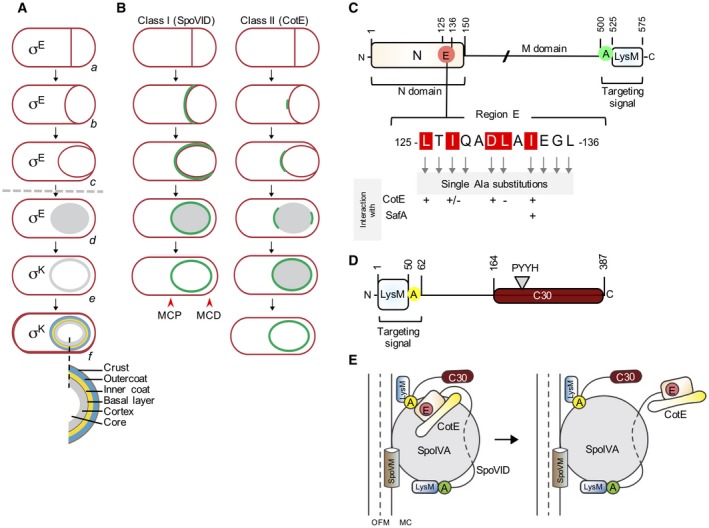
A. Stages of sporulation. The figure shows the main morphological stages of sporulation starting with asymmetric division of the rod‐shaped cell (*a*), progress through the engulfment sequence (*b* and *c*), beginning of synthesis of the spore cortex (*d*), completion of cortex synthesis (*e*), final stages in coat assembly (*f*) and spore release (g). The spore core (Cr) and the main structural layers of the mature spore are indicated in *g*. The mother cell‐specific RNA polymerase sigma factors active before and after engulfment completion (broken arrow) are indicated. B. GFP‐fusion localization patterns for early, σ^E^‐dependent, coat proteins of kinetics classes I, exemplified by SpoVID and II, represented by CotE. MCP, mother cell proximal spore pole; MCD, mother cell distal spore pole. C. Domain organization of SpoVID with the N and LysM domains represented and regions E and A highlighted. The amino acid sequence of region E is shown with the substitutions impairing the interaction of CotE shown (‘–’ sign). Only L131 was previously identified as essential for encasement by CotE and for a direct interaction with E in SpoVID. Arrows show all the single Ala substitutions used in this work. D. Domain organization of SafA showing the position of the LysM domain followed by region A, which mediates a direct interaction with SpoVID. The C30 region of the protein, produced independently through internal translation of the *safA* mRNA, is also shown, with the PYYH motif highlighted. E. Schematic view of the complex formed by SpoVM, SpoIVA, SpoVID and SafA. SpoVM recognizes the positive curvature of the outer forespore membrane (OFM, with the midplane of the membrane represented by a dashed line) and recruits SpoIVA via a direct protein‐protein interaction. SpoIVA interacts with region A close to the C‐terminal domain of SpoVID, with SafA (although the regions involved have not been mapped) and possibly with CotE (not represented for simplicity), recruiting these proteins. Encasement by all coat layers then requires interactions with the N‐terminal morphogenetic domain of SpoVID, demonstrated for SafA and CotE but postulated in the case of SpoIVA. Encasement is most likely linked to the ability of SpoIVA to polymerize in an ATP‐dependent form assisted by SpoVID, forming the coat basal layer, and also on the homomeric polymerization of SafA (and its short form C30) and CotE. MC, mother cell cytoplasm.

Endospores or spores, are metabolically dormant cellular structures that resist extreme physical and chemical parameters, and can remain viable for long periods of time (Henriques and Moran, 2007; McKenney *et al*., 2013; Driks and Eichenberger, 2016; Setlow *et al*., 2017). Sporulation begins with a polar division that forms a mother cell and a smaller forespore, the future spore (Fig. 1A). Next, the mother cell engulfs the forespore in a process analogous to phagocytosis, at the end of which the forespore becomes completely surrounded by the mother cell cytoplasm (Henriques and Moran, 2007; McKenney *et al*., 2013; Tan and Ramamurthi, 2014; Driks and Eichenberger, 2016). A thick layer of peptidoglycan named cortex, is formed between the two forespore membranes and is necessary for spore heat resistance (Henriques and Moran, 2007; McKenney *et al*., 2013; Driks and Eichenberger, 2016). The protein coat, which in *B. subtilis* consists of a basement layer, an inner coat, an outer coat and a glycoproteinaceous crust, is assembled on top of the cortex (Popham, 2002; Henriques and Moran, 2007; McKenney *et al*., 2010; McKenney *et al*., 2013; Driks and Eichenberger, 2016) (Fig. 1A). When differentiation is complete, the mother cell undergoes autolysis to relase the spore (Fig. 1A).

Coat proteins are synthesized in the mother cell under the control of a transcriptional cascade of which σ^E^, active prior to engulfment completion and σ^K^, active following engulfment completion, are the main regulators (Fig. 1A). Two steps can be distinguished in coat assembly: targeting and spore encasement (McKenney and Eichenberger, 2012; McKenney *et al*., 2013; Qiao *et al*., 2013; Driks and Eichenberger, 2016). The initial targeting of the coat proteins coincides with the onset of engulfment when the forespore membranes begin to curve. This step requires the morphogenetic protein SpoIVA. SpoIVA localizes to the forespore surface and interacts with SpoVM, an amphipatic α‐helical peptide that recognizes the positive curvature of the outer forespore membrane (Ramamurthi *et al*., 2006; Ramamurthi and Losick, 2008; Ramamurthi *et al*., 2009; Castaing *et al*., 2013). SpoIVA recruits the encasement‐promoting protein SpoVID, and three other morphogenetic proteins: SafA, essential for the assembly of the inner coat, CotE, required for outer coat assembly and CotZ, required for crust assembly and whose localization also requires CotE (Zheng *et al*., 1988; Beall *et al*., 1993; Driks *et al*., 1994; Takamatsu *et al*., 1999; Ozin *et al*., 2000; Wang *et al*., 2009; McKenney *et al*., 2010). At this stage, the early coat proteins form a cap on the spore pole that faces the mother cell cytoplasm, or mother cell proximal (MCP) pole (McKenney and Eichenberger, 2012). In the absence of SpoIVA, the coat proteins fail to localize at the spore surface and form long swirls dispersed throughout the mother cell cytoplasm (Roels *et al*., 2015; Stevens *et al*., 1992). Encasement, the phase during which the coat proteins assemble around the entire surface of the spore, is controlled by SpoVM and SpoVID, and occurs in successive waves dictated by the deployment of the mother cell transcriptional cascade (McKenney and Eichenberger, 2012; McKenney *et al*., 2013). The early coat proteins, produced under σ^E ^control, can be classified into three classes with respect to the kinetics of encasement (McKenney and Eichenberger, 2012) (Fig. 1B). Kinetics class I proteins localize to the surface of the developing spore at the onset of engulfment and track the mother cell membrane so that encasement and engulfment occur simultaneously; kinetics class II proteins localize simultaneously with class I proteins, but begin encasement only after engulfment completion from a site, at the mother cell distal (MCD) pole, where fission of the engulfing membranes occurred (Fig. 1B); kinetics class III proteins localize simultaneously with class I and class II proteins, but begin encasement when phase dark forespores first appear (McKenney and Eichenberger, 2012). The late coat proteins, produced under the control of σ^K^, begin encasement just after engulfment completion (kinetics class IV), or when the forespore becomes phase dark (class V) or phase bright (class VI) (McKenney and Eichenberger, 2012).

SpoVID has an N‐terminal domain (N) that resembles the coat protein of phage PP7. The N domain is followed by a middle domain (M) with a possibly extended conformation. The C‐terminal end of the protein is characterized by the presence of a targeting signal formed by a 24 residue stretch called region A, followed by a LysM domain (Beall *et al*., 1993; Ozin *et al*., 2001b; Costa *et al*., 2006; Wang *et al*., 2009) (Fig. 1C). It is region A that mediates the interaction with SpoIVA, which, together with a less important contribution from the LysM domain, brings SpoVID to the forespore outer membrane (Costa *et al*., 2006; Wang *et al*., 2009). SafA and most likely CotE interact directly with SpoIVA but the region involved in this interaction has not been identified (Mullerova *et al*., 2009; Qiao *et al*., 2012) (Fig. 1D). Thus, the targeting step relies on a series of interactions with SpoIVA that result in the formation of an organizational scaffold for encasement (Mullerova *et al*., 2009; Wang *et al*., 2009; Qiao *et al*., 2012) (Fig. 1E).

The N domain of SpoVID is essential for encasement by all proteins whose localization has been studied using GFP fusions, including SpoIVA (Wang *et al*., 2009; de Francesco *et al*., 2012; McKenney and Eichenberger, 2012). SpoIVA assembles into cables in an ATP‐dependent manner (Ramamurthi and Losick, 2008; Castaing *et al*., 2013), forming the basal layer on top of which the rest of the coat is built. A second, as yet unidentified, interaction between SpoIVA and the N domain of SpoVID is likely to occur (Mullerova *et al*., 2009; de Francesco *et al*., 2012). Polymerization of SpoIVA may be restricted to the spore surface and may be facilitated by SpoVID during encasement (McKenney and Eichenberger, 2012; Castaing *et al*., 2013; McKenney *et al*., 2013; Driks and Eichenberger, 2016). Encasement by SafA and the SafA‐dependent proteins, as well as encasement by CotE and the CotE‐dependent coat proteins, requires an interaction with the N‐domain of SpoVID (Costa *et al*., 2006; Mullerova *et al*., 2009; de Francesco *et al*., 2012; McKenney and Eichenberger, 2012; Qiao *et al*., 2012; 2013) (Fig. 1C). SafA is a proline‐rich modular protein that exists in its full‐length form of 45 kDa, SafA^FL^ and a 30 kDa form named C30 that results from internal translation of the *safA* transcript (Takamatsu *et al*., 1999; Ozin *et al*., 2001a; 2001a). SafA bears a localization signal close to its N‐terminus, formed by a LysM domain, followed by a 12 amino acids residues region A that contacts the N domain of SpoVID (Costa *et al*., 2006) (Fig. 1D). This interaction is essential for the localization of SafA at the cortex/inner coat interface (Ozin *et al*., 2000; Ozin *et al*., 2001a; Costa *et al*., 2006; Fernandes *et al*., 2018). It is SafA^FL^ that is essential for inner coat assembly (Ozin *et al*., 2000; Ozin *et al*., 2001a) and recruits C30 to the spore surface. Both SafA^FL ^and C30 self‐interact (Ozin *et al*., 2000). Multimerization of SafA^FL ^and C30 may be promoted by the interaction of SafA^FL ^with SpoVID during encasement (de Francesco *et al*., 2012; Ozin *et al*., 2000). CotE also appears to have a modular design, with different regions of the protein implicated in localization, homo‐oligomerization and binding to other coat proteins (Bauer *et al*., 1999; Kim *et al*., 2006). CotE is found at the inner coat/outer coat interface, where it nucleates assembly of the outer coat (Driks *et al*., 1994). Similar to SafA, CotE forms large homo‐oligomers (Aronson *et al*., 1992; Costa *et al*., 2006; Krajcikova *et al*., 2009), a property that may be controlled by SpoVID during encasement.

In a *safA *null mutant, the inner coat proteins are not recruited to the spore surface and, similarly, the outer coat is not formed in a *cotE *null mutant (Zheng *et al*., 1988; Takamatsu *et al*., 1999; Ozin *et al*., 2000; Costa *et al*., 2006; McKenney and Eichenberger, 2012). Thus, the inner and outer coat layers appears to assemble largely independently (Henriques and Moran, 2007; McKenney *et al*., 2013; Driks and Eichenberger, 2016). Yet, recent work has shown that a short stretch of 12 amino acids, termed E for encasement, close to the end of the N domain of SpoVID, is essential for encasement by all layers of the coat (de Francesco *et al*., 2012). As in a *spoVID *deletion mutant, deletion of E blocks encasement at the single cap stage. This coat cap eventually detaches from the spore surface and large horseshoe‐shaped pieces that accumulate in the mother cell cytoplasm can be observed by TEM (Beall *et al*., 1993; Wang *et al*., 2009). Single amino acid substitutions, L125A, L127A and I131A, within region E of SpoVID prevent encasement by CotE and CotE‐dependent outer coat proteins. Furthermore, I131A and L127A impaired the interaction with CotE, implying that encasement by CotE and its partners rely on an interaction with SpoVID, via E (de Francesco *et al*., 2012). L125A, however, did not impair the interaction with CotE, indicating that this substitution affected encasement by some other mechanism. Residues specifically affecting the interaction of SafA with SpoVID, and encasement by the inner coat, have not been identified.

We now identify residues within region E that are essential for the interaction with, and encasement by, SafA and SafA‐dependent proteins. We show that the L125A and L127A substitutions prevent binding of SafA to SpoVID and encasement by SafA. The specificity of the L125A substitution suggests that mislocalized SafA and other inner coat proteins act as an attractor for CotE and its partners. Although the residues with critical roles in the interactions with SafA and CotE are overlapping, we provide evidence that SpoVID acts essentially as a non‐competitive hub in the coordination of inner and outer coat encasement. A key mechanism underlying this behavior may be that the interaction of SafA with E allows it to establish a second interaction with SpoVID, possibly releasing SafA from E.

## Results

### Encasement kinetics suggest that SafA is a kinetics class II protein

SafA was not included in the original study of McKenney and Eichenberger (McKenney and Eichenberger, 2012), where the kinetics of encasement by different coat proteins was defined, as the available SafA‐GFP fusion protein was non‐functional. Here, we used a *safA‐fl3‐yfp *fusion which differs from the non‐functional *safA‐gfp *in that SafA is separated from YFP by a flexible linker formed by three repeats of the GGGGS sequence (Arai *et al*., 2001) (Fig. S1A). As judged from the profile of extractable coat proteins resolved by SDS‐PAGE and by spore resistance and germination assays (Fig. S1B–D), *safA‐fl3‐yfp *efficiently complements the phenotypes caused by a *safA *in frame‐deletion mutation, obtained through integration followed by resolution of a non‐replicative plasmid ((Arnaud *et al*., 2004); see also the Supporting Material and Methods). Therefore, we used SafA‐FL3‐YFP, abbreviated to SafA‐YFP for simplicity, as a reporter for studying encasement by SafA.

**Figure 2:**
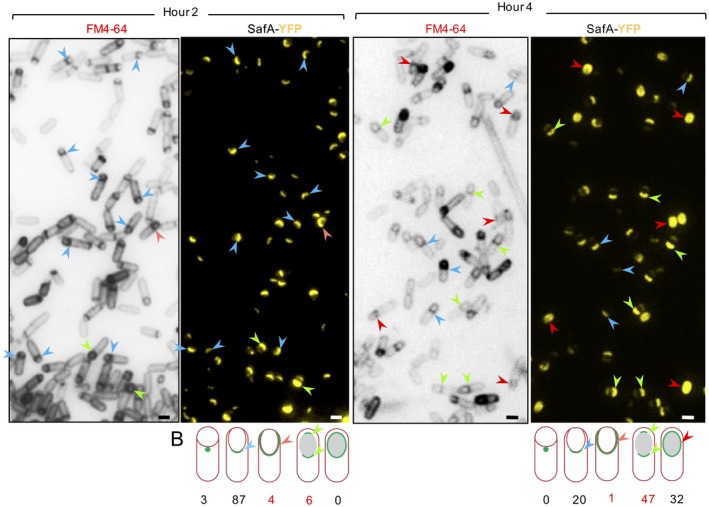
SafA is a kinetic class II protein*. *A. The localization of SafA‐YFP was monitored in a WT background at the indicated times in hours after the onset of sporulation in SM medium. Culture samples were collected at hour 2 and 4 after the onset of sporulation, stained with FM‐4‐64 and analyzed by fluorescence microscopy (see also Fig. S2). The FM4‐64 images are shown in black for improved contrast. Arrowheads are defined as follows: blue, the single cap at the MCP pole; green, the two‐cap pattern (note the weaker fluorescence intensity at the MCD pole); red, SafA‐YFP surrounding the entire surface of the spore; pink, a kinetics class I pattern (SafA‐YFP tracking the engulfment membranes). Scale bar, 1 µm. B. The percentage of sporangia showing the localization pattern represented is indicated for the samples analyzed at hour 2 and hour 4 of sporulation (see also Fig. S2).

To determine the kinetics class of SafA‐YFP, a ∆safA strain expressing *safA* under the control of P*_safA_* at the non‐essential *amyE* locus, was induced to sporulate by ressuspension into Sterlini‐Mandelstam medium (SM) (Sterlini and Mandelstam, 1969). Samples were collected 2 and 4 h after resuspension, stained with the membrane impermeable dye FM4‐64 and examined by phase contrast and fluorescence microscopy. FM4‐64 does not cross membranes, and therefore, is unable to stain the forespore membranes following fission of the engulfing membranes upon engulfment completion. Sporangia at intermediate stages in engulfment can thus be distinguished from those where engulfment has been completed (Pogliano *et al*., 1999). At hour 2 of sporulation, SafA‐YFP localizes as a single cap at the mother cell proximal (MCP) spore pole in 91% of the sporangia scored (Fig. 2 and S2; Table S1). The cap was curved, consistent with initiation of the first phase of encasement at the onset of engulfment (McKenney and Eichenberger, 2012). Of the sporangia with a single cap, 87% showed an expanded cap which did not accompany the engulfing membranes (Fig. 2, blue arrowheads), a phenotype distinct from typical kinetics class I proteins, for which encasement occurs concomitantly with engulfment (McKenney and Eichenberger, 2012). Nearly, complete rings of YFP fluorescence were observed that overlapped with nearly complete rings of FM4‐64 fluorescence in about 4% of the sporangia (Fig. 2, pink arrowheads and Fig. S2). At hour 4 of sporulation, SafA‐YFP is seen as two unconnected caps covering both poles of the spore in 47% of the sporangia in which engulfment was completed, as judged from the lack of the FM4‐64 signal around the forespore (Fig. 2, green arrowheads and Fig. S2; Table S1). At hour 4, SafA‐YFP was also found around the entire spore in 32% of the sporangia, a pattern that increased to 83% by hour 6 (Fig. 2, red arrowheads, and Fig. S2; Table S1). Previously, SafA was proposed to be a kinetics class I protein because it interacts with the kinetics class I protein SpoVID and because the SafA‐dependent fusions YaaH‐GFP and YuzC‐GFP showed class I kinetics (McKenney and Eichenberger, 2012; but see below). The functional SafA‐YFP fusion, however, did not track the engulfing membranes in most of the sporangia scored, and thus in our hands, it exhibited the encasement pattern associated with kinetic class II proteins (McKenney and Eichenberger, 2012).

### Residues L125, I127 and I133 in region E of SpoVID are required for encasement by SafA

A previous study, in which five residues within region E (residues 126‐135 of SpoVID) conserved among SpoVID orthologues were individually substituted by Ala, has shown that Leu125 (L125), Ile127 (I127) and Leu131 (L131) are essential for spore encasement by CotE and CotE‐dependent proteins (de Francesco *et al*., 2012) (Fig. 1C). The L131A substitution and to a lesser extent, I127A, also impaired the interaction of CotE with SpoVID, indicating that this interaction is required for encasement (de Francesco *et al*., 2012). In contrast, L125A did not affect the interaction of CotE with SpoVID, suggesting that the mutation blocked encasement by some other mechanism (de Francesco *et al*., 2012) (Fig. 1C). L131A also did not impair the interaction of SafA with SpoVID suggesting that residues other than L131, perhaps, including L125, are important for this interaction (de Francesco *et al*., 2012). We reasoned that further alanine‐scanning mutagenesis of region E could reveal residues specifically required for the interaction with SafA.

We expanded the previous Ala‐scanning analysis of E (de Francesco *et al*., 2012) to all non‐Ala residues within this region, for a total of 10 single Ala substitutions (Fig. 1C, grey arrows). Additionally, we constructed an in‐frame deletion mutant of *spoVID *as well as a mutant bearing an in‐frame deletion of region E (see the supporting information). We then analyzed assembly of SafA‐YFP in ∆*spoVID *and *spoVID* ^∆E^ mutants, as well as in the mutants with single Ala substitutions in region E. To determine whether the various forms of SpoVID accumulated in the cell, we constructed strains bearing an in‐frame deletion of *spoVID *(Δ*spoVID*) and either *spoVID^WT^* or the various region E alleles, including an in‐frame deletion of the entire region E (*spoVID* ^∆E^) at *amyE*. We first verified that *spoVID^WT^* at *amyE *complemented the Δ*spoVID* mutation (Table 1; see also the Supporting information). We then used immunoblotting with an anti‐SpoVID antibody (Ozin *et al*., 2000) to monitor the accumulation of the various SpoVID forms at hours 2, 4 and 6 after the onset of sporulation. These experiments revealed that all forms of SpoVID were produced at significant levels during sporulation (Fig. S3). SpoVID^L125A^, however, was less abundant than all other SpoVID variants at hours 2 and hour 6, possibly because of some instability at early and late times of sporulation (Fig. S3).

**Table 1:** Heat and lysozyme resistant spore counts.

	Titer of spores (CFU ml^–1^)^a^									
	WT	WT^C^ ^2^	*∆spoVID* b	*∆safA*	*∆cotE*	∆E^c^	L125A^c^	I127A^c^	L131A^c^	I133A^c^
Viable	1.8±0.4 × 10^8^	2.2±0.3 × 10^8^	8.0±1.6 × 10^7^	1.6±0.5 × 10^8^	1.3±0.2 × 10^8^	6.2±0.7 × 10^7^	5.2±0.8 × 10^7^	6.6±1.3 × 10^7^	2.2±0.3 × 10^8^	1.9±0.2 × 10^8^
Heat	1.7±0.2 × 10^8^	1.9±0.4 × 10^8^	3.7±1.5 × 10^7^	1.7±0.4 × 10^8^	9.9±2.9 × 10^7^	3.4±0.5 × 10^7^	5.4±5.1 × 10^7^	6.3±4.4 × 10^7^	1.9±0.2 × 10^8^	1.8±0.3 × 10^8^
Lysozyme	1.7±0.3 × 10^8^	2.0±0.2 × 10^8^	3.6±1.0 × 10^7^	7.4±2.5 × 10^7^	4.5±1.9 × 10^7^	1.8±0.7 × 10^6^	3.2±1.8 × 10^6^	1.0±0.7 × 10^7^	1.9±0.3 × 10^8^	1.6±0.2 × 10^8^

a the numbers represent the average and standard deviation of three different experiments (see the Material and Methods section). CFU, colony‐forming units.

b WT^C^ denotes a strain with an in‐frame deletion of *spoVID *and a WT *spoVID *allele inserted at the non‐essential *amyE *locus.

c all strains have an in frame deletion of *spoVID *and the indicated alleles inserted at *amyE*: a complete deletion of region E (ΔE) and single alanine substitutions of residues L125, I127, L131 and I133*.*

**Figure 3:**
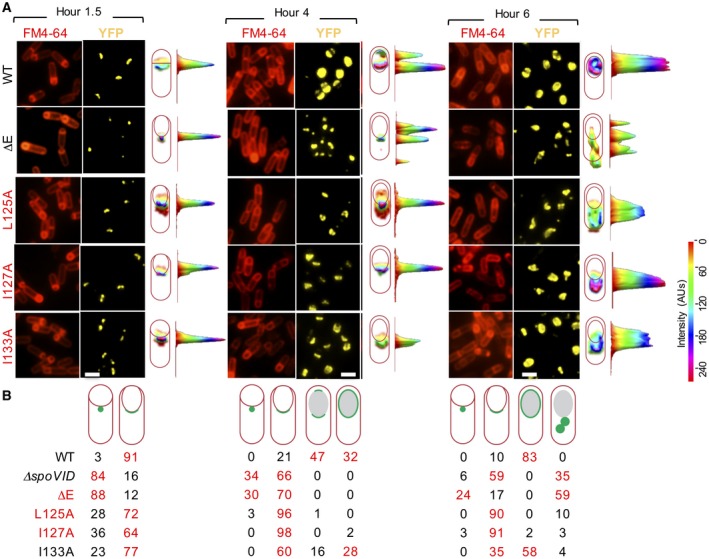
Identification of residues within region E that affect the localization of SafA‐YFP*. *A. The localization of SafA‐YFP was monitored throughout sporulation in the WT or in strains bearing the indicated mutations in *spoVID. *Culture samples were collected at hours 1.5, 4 and 6 after the onset of sporulation by in SM medium, stained with FM‐4‐64 and analysed by fluorescence microscopy (see also Fig. S2). One cell representative of the prevalent pattern at each time point was choosen to show the distribution of the fluorescence signal in three dimensional intensity graphs. Scale bar, 1 µm. B. The percentage of sporangia showing the localization pattern represented schematically is indicated for the various strains at the different sampling times (see also Table S1).

The localization of SafA‐YFP was then monitored in parallel for the WT and the various *spoVID *mutants during sporulation induced by resuspension. The results for the WT and the ΔE, L125A, I127A and I133A mutants are shown in Fig. 3, the complete panel of mutants is included in Fig. S2 and the quantitative analysis of the patterns observed is show in Table S1. For this group of experiments, ΔE and the various point mutations in E were introduced at the wild type *spoVID *locus of a Δ*safA *mutant through integration and resolution of a non‐replicative plasmid, as described above for the construction of the *safA* in‐frame deletion mutant ((Arnaud *et al*., 2004); see also the Supporting Material and Methods). In those strains, *safA‐yfp *was expressed from the *amyE *locus. SafA‐YFP localized as a single focus (dot) on the MCP pole in ∆*spoVID *(in 84% of the sporangia) and *spoVID* ^∆E^ (in 88% of the sporangia) at hour 2 of sporulation (Figs. 3 and S2; Table S1). At hour 4, the pattern seen at hour 2 persisted in about 30% of the sporangia. In 66% of ∆*spoVID *sporangia and 70% of *spoVID* ^∆E^ sporangia, SafA‐YFP accumulated on the MCP pole, forming an irregular cap (Fig. 3). Neither complete encasement of the forespore, nor the formation of a second cap at the MCD pole, were observed, even at hours 6 and 8 (Figs. 3 and S2; Table S1). Moreover, at hours 6 and 8, the fluorescence signal was found dispersed throughout the mother cell cytoplasm in 35% of ∆*spoVID *sporangia and in 59% of *spoVID* ^∆E^ sporangia, suggesting that SafA‐YFP detached from the forespore (Fig. 3). Thus, in ∆*spoVID *and *spoVID* ^∆E^ mutants, SafA‐YFP can localize at the surface of the forespore as a dot on the MCP pole, but does not form a cap, in agreement with earlier work (Wang *et al*., 2009). SpoVID is therefore required for expansion of the SafA‐YFP dot into the cap that in the WT covers the exposed surface of the forespore during the initial stages of engulfment.

We then examined the effect of the various Ala substitutions on encasement by SafA‐YFP. The WT pattern of SafA‐YFP localization was observed for mutants with the T126A, Q128A, D130A, L131A, E134A, G135A or L136A substitutions (Figs. 3 and S2; Table S1). D130A and L131A were also included in the screen conducted by de Francesco and co‐authors (de Francesco *et al*., 2012). While the D130A substitution had no effect on encasement by CotE‐GFP, L131A impaired encasement by CotE‐GFP and by proteins of all coat layers, including the SafA‐dependent YaaH‐GFP (de Francesco *et al*., 2012). As mentioned above, this result was surprinsing given that L131A reduced, but did not eliminate, the interaction of CotE with SpoVID and had no effect on the interaction of SafA with SpoVID (de Francesco *et al*., 2012) (see also below). The effect of L131A on encasement was attributed to a possible impaired interaction with the basal layer protein SpoIVA (de Francesco *et al*., 2012). We don´t presently know why in our hands L131A shows no effect on SafA‐YFP, which implies that encasement by SpoIVA must also be largely unaffected, but we note that our observation is consistent with the lack of effect of L131A on the interaction of SafA with SpoVID ((de Francesco *et al*., 2012); above).

We found encasement to be affected by the L125A and I127A and I133A single amino acid substitutions. At hour 1.5 of sporulation, SafA‐YFP formed a dot (in 28% of the sporangia for L125A, 36% for I127A, and 23% for I133A) or a cap on the MCP pole (in 72% of the sporangia for L125A, 64% for I127A, and 77% for I133A) (Fig. 3 and Table S1). By hour 4, very few if any dots were seen, and caps at the MCP pole were the predominant pattern (in 96% of the sporangia for L125A, 98% for I127A and 60% for I133A). By hour 6, the cap at the MCP pole pattern was observed in 90% of the L125A sporangia and in 91% of the I127A sporangia (Fig. 3). A second cap or complete encasement of the spore were almost never observed for the L125A and I127A strains at hours 4 or 6 of sporulation (Fig. 3). The I133A strain differed, however, in that a cap at the MCD spore pole (hour 4, 16%; hour 6, 35%) or complete encasement of the forespore (hour 4, 28%; hour 6, 58%) were detected in a significant number of cells (Fig. 3). In the WT, complete encasement was detected for 32% of the sporangia at hour 4 and for 83% at hour 6 (Fig. 3 and Table S1). Thus, L125A, I127A and to a lesser extent I133A delayed formation of the MCP cap; L125A and I127A, however, but not I133A, also prevented formation of the MCD cap.

### The L125A and I127A substitutions in E impair encasement by both a SafA‐dependent protein and a CotE‐dependent protein

Since L125A and L127A impaired, while I133A delayed, encasement by SafA‐YFP, we next wanted to test whether these substitutions also affected encasement by a SafA‐dependent protein. In addition, L125A and I127A (and L131A; above) were reported to prevent encasement by a CotE‐dependent protein, while only L131A affected the CotE‐SpoVID interaction (de Francesco *et al*., 2012). Here, we analyzed encasement by YaaH‐GFP (SafA‐dependent) and CotM‐GFP (CotE‐dependent) produced from their native locus, in the ΔE and L131A strains, as well as the *spoVID^L125A^*, *spoVID^I127A^* and *spoVID^I133A^*alleles. YaaH‐GFP was previously classified as kinetics class I protein, whereas CotM‐GFP was assigned to kinetic class II (McKenney and Eichenberger, 2012). In the WT, YaaH‐GFP formed a cap at the MCP pole (in 82% of the sporangia at hour 2 of sporulation) that did not track the engulfing membranes (Fig. S4A; see also Fig. S5 for larger fields of cells; quantification in Table S2). A second cap, at the MCD pole, appeared after engulfment completion (in 47% of the sporangia at hour 4) and complete encasement was detected between hours 4 (36% of the sporangia) and hour 6 (in 75% of the sporangia) (Figs. S4 and S5; Table S2). Thus, like SafA‐YFP, YaaH‐GFP behaved, in our hands, like a kinetics class II protein.

YaaH‐GFP showed the same genetic requirements for encasement as SafA‐YFP: encasement was impaired in *spoVID *and *spoVID^∆E^* sporangia, as well as in *spoVID^L125A^*and *spoVID^I127A^* sporangia, as seen before (de Francesco *et al*., 2012) (Fig. S4A). However, although these authors reported that L131A blocked encasement by YaaH‐GFP, we did not observe a similar effect in our study (Fig. S4A; Table S2), consistent with our observation that L131A did not impair encasement by SafA‐YFP (see above). Finally, I133A which delayed encasement by SafA‐YFP, also seemed to delay encasement by YaaH‐GFP; the two cap pattern represented 47% of the WT sporangia at hour 4 of sporulation but only 5% for the mutant (Fig. S4A and Table S2). However, the transition from the two cap pattern to complete encirclement of the forespore appeared faster than in the WT (Fig. S4A and Table S2). YaaH (also known as SleL), most likely a peptidoglycan hydrolase required for spore germination, has two N‐terminal LysM domains (Kodama *et al*., 1999; Lambert and Popham, 2008) and it is tempting to speculate that binding to the cortex or peptidoglycan precursors facilitates encasement completion. Thus, the substitutions, L125A, I127A, that more drastically affect encasement by SafA‐YFP, also delay spore encasement by YaaH‐GFP, consistent with the role of SafA in governing encasement by inner coat proteins (McKenney and Eichenberger, 2012).

CotM‐GFP initially localized as a dot at the MCP pole, then formed a cap and, following engulfment completion, it formed a second cap at the MCD pole from where it encases the spore, consistent with class II kinetics and its dependency on CotE (Fig. S4B and Table S3) (McKenney and Eichenberger, 2012). The L125A, I127A and L131A substitutions, that impair encasement of CotE‐GFP, also blocked encasement by CotM‐GFP (Fig. S4B and Table S3) (McKenney and Eichenberger, 2012). The delay in SafA‐YFP and YaaH‐GFP encasement caused by I133A was not observed for CotM‐GFP. In contrast, at least under our conditions, the effect of L131A was specific for CotM‐GFP, and supports the inference that the effect of L125A on encasement by outer coat proteins is indirect.

### The L125A, I127A and I133A substitutions impair the interaction of SafA with SpoVID

**Figure 4:**
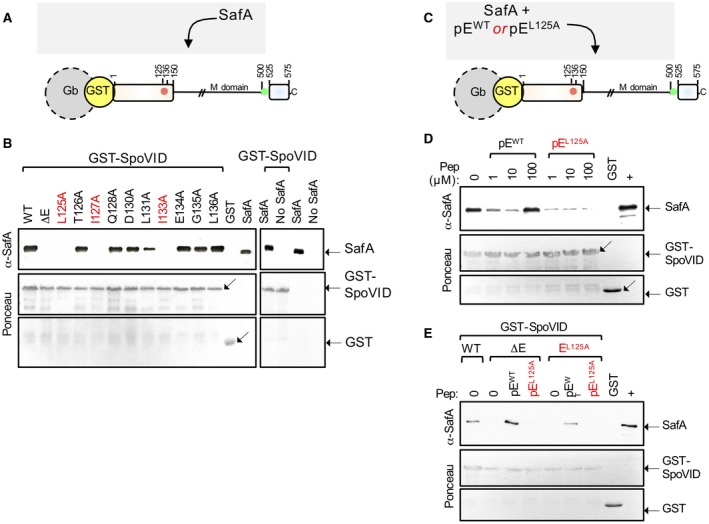
Role of region E in the interaction of SafA with SpoVID. A. design of the pull‐down assay. GST‐SpoVID fusion proteins were bound to glutathione beads (Gb) and incubated with extracts from an *E. coli* BL21(DE3) strain overproducing SafA. B. GST‐SpoVID or forms of the fusion protein with a deletion of region E (ΔE) or the indicated single alanine substitutions in region E were used as baits; GST was also immobilized, as a negative control (left panel, lane labeled ‘GST’). Following washing, bound proteins were eluted, resolved by SDS‐PAGE and the gels subject to immunoblot analysis using an anti‐SafA antibody (Ozin *et al*., 2000). As a control for the specifity of the antibody, SafA partially purified from *E. coli* was also loaded (left panel, last lane). In addition, immobilized GST‐SpoVID was incubated with extracts from *E. coli* cells that were induced (right panel, first lane) or not (second lane), to produce SafA; the two extracts were also direclty applied to the gel (third and fourth lanes). C. Region E synthetic peptides, with the WT sequence (pE^WT^) or with the L125A substitution (pE^L125A^), were incubated with extracts from an *E. coli* strain that overproduces SafA. The mixture was then incubated with GST‐SpoVID immobilized on glutathione beads. Following washing, bound proteins were eluted, resolved by SDS‐PAGE and subject to immunoblotting with anti‐SafA antibodies to assess binding to GST‐SpoVID. D. The assay was conducted with the pE^WT^ and pE^L125A^ peptides at the indicated concentrations. E. the extract from the *E. coli* strain that produces SafA was incubated in the absence (‘0’) or in the presence of peptides pE^WT^ or pE^L125A^ at a final concentration of 1 mM, and the mix was then incubated with immobilized GST‐SpoVID^WT^ (WT), GST‐SpoVID^ΔE^ (ΔE) or GST‐SpoVID^L125A^ (L125A). In D and E: GST alone was bound to the glutathione beads as a negative control (lane marked GST). Partially purified SafA was loaded in the lane marked with the sign ‘+’ as a positive control for the antibody. In B, D and E, the membranes were stained with Ponceau red to control for the amount of GST‐SpoVID (middle panels) bound to the beads or for the presence of GST (bottom panels).

We then wanted to test which residues in E contributed to an interaction with SafA. We fused a GST tag to the N‐terminus of SpoVID^WT ^or SpoVID^∆E^, or to the versions of the protein with the L125A, I127A and I133A substitutions, as well as all other substitutions included in our Ala scanning (Fig. 4A). The various GST‐SpoVID proteins were overproduced in *Escherichia coli*, bound to glutathione beads and incubated with extracts from an *E. coli* strain producing SafA. Following washing of the beads, the eluted proteins were resolved by SDS‐PAGE and subjected to immunoblot analysis with an anti‐SafA antibody (Ozin *et al*., 2000) (Fig. 4B). As expected, GST‐SpoVID^WT ^was able to pull‐down SafA from the extracts (Ozin *et al*., 2000; Mullerova *et al*., 2009) (Fig. 4B). Fusions of GST to SpoVID^T126A^, SpoVID^Q128A^, SpoVID^D130A^, SpoVID^E134A^, SpoVID^G135A^ and SpoVID^L136A^, also pulled down SafA from the extracts (Fig. 4B), indicating that these residues are dispensable for the interaction. GST‐SpoVID^∆E^, SpoVID^L125A^, SpoVID^I127A^ and SpoVID^I133A^, however, were unable to pull‐down SafA (Fig. 4B). We conclude that the side chains of L125, I127 and I133 in E play an important role in the interaction between SpoVID and SafA. In our pull‐down assay, retention of SafA by GST‐SpoVID^L131A^ was slightly reduced (Fig. 4B). Plausibly, L131 contributes to the interaction with SafA, though to a lesser extent than L125, I127 or I133. These results suggest that the defect caused by L125A, I127A and I133A on encasement by SafA is due to an impaired interaction with SpoVID. L125A and I127A also reduced the pull down of CotE by immobilized SpoVID (de Francesco *et al*., 2012). Thus, within region E, two residues, L125 and I133, are specifically required for the interaction of SafA with SpoVID. Two other residues, I127 and L131A, contribute to the interaction of both SafA and CotE with SpoVID.

### Region E is sufficient for binding of SafA to SpoVID

We then wanted to test whether region E of SpoVID is sufficient for the interaction with SafA. We started by testing the ability of region E peptides to compete for SafA binding to GST‐SpoVID in pull‐down assays (Fig. 4C). These assays were designed as described in the preceding section, except that the extracts prepared from *E. coli *cells producing SafA were incubated with a synthetic biotinylated peptide with the sequence of region E (pE^WT^) prior to addition to immobilized GST‐SpoVID^WT^. In parallel, the extracts from the SafA‐producing *E. coli* were pre‐incubated with a similar peptide but carrying the L125A substitution (pE^L125A^) (Fig. 4B).

Pre‐incubation of the SafA‐containing extracts with pE^WT^ at concentrations of 1 and 10 µM, reduced the retention of SafA by immoblized GST‐SpoVID^WT^ as compared to a control assay in which pE^WT^ was not added (Fig. 4D). Thus, pE^WT^ appears to compete with SpoVID for binding to SafA, in line with the idea that the direct interaction between the two proteins occurs through E. At a concentration of 100 µM, however, pE^WT^ increased the retention of SafA by immobilized GST‐SpoVID^WT^ (Fig. 4D). One interpretation of this result is that binding of SafA to E may facilitate an interaction of the protein with a second region of SpoVID. To test whether the peptides bound to SafA, the experiments were repeated but the peptides were incubated with the *E. coli *extracts producing GST‐SpoVID^WT^ before its immobilization in glutathione beads; the mixture was then washed before addition of the SafA extract. Under these conditions, no effect of the peptides was seen in the retention of SafA by GST‐SpoVID^WT^. This suggests that the peptides bound to SafA and not to SpoVID (not shown). pE^L125A^ reduced, but did not eliminate retention of SafA by GST‐SpoVID^WT^ at 1, 10 µM or 100 µM (Fig. 4D). At least in vitro, pE^L125A^ may still bind to SafA but is unable to promote its binding to a second region of SpoVID.

### Bypass of region E for the interaction of SafA with SpoVID

We then tested whether the incubation of pE^WT^ but not pE^L125A^, could bypass the requirement for residue L125 in binding of SafA to GST‐SpoVID. We incubated the *E. coli* extracts with SafA with 100 µM of pE^WT^ or pE^L125A ^(Fig. 4E). The SafA‐containing extracts were then tested for retention by immobilized GST‐SpoVID^WT^ and GST‐SpoVID^∆E^; the SafA extracts were additionally tested against GST‐SpoVID^L125A^.

When pre‐incubated with pE^WT^, SafA was pulled‐down by GST‐SpoVID^WT^, by GST‐SpoVID^∆E^ and by GST‐SpoVID^L125A^ (Fig. 4E). In contrast, the pre‐incubation of SafA with pE^L125A^ did not result in its retention by GST‐SpoVID^∆E^ or by GST‐SpoVID^L125A^ (Fig. 4E). These results also suggest a model in which binding of SafA to E, promotes binding of SafA to a second region of SpoVID.

### The L125A, I127A and I133A substitutions affect the distribution of SafA in mature spores

**Figure 5:**
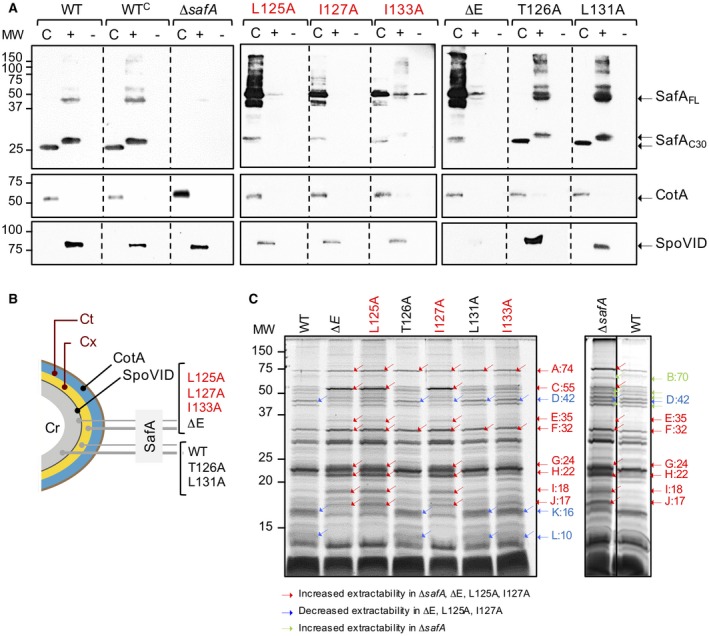
A. Single amino acid substitutions L125A, I127A and I133A affect the composition of the spore surface layers. A. spores were produced in SM medium, collected 18 h after resuspension and density‐gradient purified. Spores were from WT cultures or cultures of a Δ*spoVID* mutant producing either the WT (WT^C^) or the indicated forms of SpoVID from *amyE. *The spores were decoated to produce a coat fraction (C); the decoated spores were then re‐extracted before (‘–’) or after (‘+’) treatement with lysozyme (Fernandes *et al.*, 2018). Proteins present on the different fractions were resolved by SDS‐PAGE and immunobloted with anti‐SafA, anti‐CotA and anti‐SpoVID antibodies. B. diagram summarizing the localization of SafA, CotA and SpoVID in the various layers of the spore, as deduced from the analysis in A. Cr: spore core; Cx: cortex; Ct: coat. The thicker grey lines denote higher representation of SafA in the indicated spore layers. C Spores from the indicated strains, incubated for 24 h in resuspension medium, were density‐gradient purified, the coat proteins extracted and resolved by SDS‐PAGE. The gels were stained with Coomassie brilliant blue. Protein bands are identified by letters (A to L), followed by their apparent molecular weight, in kDa. Bands that show increased extractability in Δ*safA*, ΔE, L125A and I127A spores relative to the WT are shown in red; bands showing reduced extractability from ΔE, L125A and I127 spores are shown in blue; bands showing increased extractability from Δ*safA* spores are highlighted in green. The position of molecular weight markers (in kDa) are show on the left side of the panels.

We have shown recently that SafA is found both in the coat and in a cortex fraction (Fernandes *et al*., 2018) and it seemed possible that region E mutations, in particular *spoVID^∆^^E^*, *spoVID^L125A^* and *spoVID^I127A^* could alter the distribution of SafA in mature spores. Spores were purified by density gradient centrifugation, decoated by SDS/DTT extraction to produce a coat fraction, and the decoated spores were re‐extracted before or after incubation with lysozyme; treatment of the decoated spores with lysozyme produced a ‘cortex’ fraction while the re‐extraction of the decoated spores with no prior lysozyme treatment served as a control for the effect of lysozyme (Fernandes *et al.*, 2018). Proteins in these fractions were resolved by SDS‐PAGE and subject to immunoblot analysis with an anti‐SafA antibody. Since SpoVID^∆E^ or the Ala‐substituted forms were produced from *amyE* in a *∆spoVID* mutant, we also assayed WT spores, and spores of a *∆spoVID* mutant complemented by *spoVID* ^WT^ at *amyE* (WT^C^ in Fig. 5A). Spores of a strain producing the T126A form of SpoVID, which does not impair the SafA‐SpoVID interaction, were also included in the analysis as a negative control. In WT or WT^C^ spores, full‐length SafA and several multimeric forms of the protein were found in the cortex fraction, whereas the C30 form of SafA was found in both the cortex and coat fractions (Fig. 5A), in agreement with previous results (Fernandes *et al.*, 2018). C30, extracted from the cortex migrated slower that the form of the protein released from the coat, as also observed before (Fernandes *et al.*, 2018) (Fig. 5A). A possibility is that C30 is processed by the YabG protease in the coat but not in the cortex (Fernandes *et al.*, 2018). To test this idea, we examined the localization of SafA in the different spore fractions in spores produced by a *yabG *insertional mutant. No C30 was found in the cortex fraction of *yabG *spores (Fig. S6, top panel). The apparent size of C30 found in the coat of *yabG *spores, however, migrated slower than the C30 form found in the coat of WT spores but slightly faster than the form found in the cortex (Fig. S6). We do not presently know why C30 is absent from the cortex of *yabG *spores. In any event, this observation is consistent with processing of C30 by YabG in the coat of WT spores, and further suggests that C30 in the cortex may be cross‐linked to a small protein or to the cortex peptidoglycan (Fernandes *et al.*, 2018).

The mutants formed two groups (Fig. 5A): (i) in spores of the *spoVID^∆^^E^*, *spoVID^L125A^*, *spoVID^L275A^* and *spoVID^I133A^* mutants, the extractability of full‐length SafA as well as the extractability of the multimeric forms of the protein, increased significantly in the coat fraction; C30 was weakly extracted from the coat, it showed the apparent mass of the cortex‐associated protein found in WT extracts, and was not extractable from the cortex; (ii) in *spoVID^T126A^* and *spoVID^I131A ^*mutants the distribution of the different forms of SafA was very similar to the WT, except that more multimeric forms of the protein were released from the cortex.

In all strains, SpoVID was only detected in the cortex fraction, and except for the T126A strain, its levels did not differ much among the various strains (Fig. 5A). In contrast, CotA, a well‐characterized coat protein was only detected in the coat fraction (Donovan *et al*., 1987; Martins *et al*., 2002; Fernandes *et al.*, 2018) (Fig. 5A). Thus, the Ala substitutions that had the greatest impact on the localization of SafA‐YFP and that impaired the interaction of SafA with SpoVID also most affected the distribution of SafA in mature spores (Fig. 5B). These results indicate that the final distribution of SafA in mature spores relies on its interaction with SpoVID, established during encasement.

### Region E mutations affect the composition and properties of the spore

Deletion of *safA* leads to the production of spores that are heat resistant but susceptible to lysozyme (Takamatsu *et al*., 1999; Ozin *et al*., 2000). Heat resistance is a spore property dependent on proper assembly of the cortex, whereas the coat confers resistance to lysozyme by preventing access to the cortex (Henriques and Moran, 2007; McKenney *et al*., 2013; Setlow, 2014). To test whether the Ala substitutions in region E that cause mislocalization of SafA, specifically compromised a coat‐related spore property, the total viable cell count, as well as the heat‐resistant and the lysozyme‐resistant cell count were measured for the WT and the various mutants, 48 h after the onset of sporulation (Table 1). The viable, heat‐ and lysozyme‐resistant cell counts were nearly identical for the WT (1.8 ± 0.4 × 10^8^/1.7 ± 0.2 × 10^8^/1.7 ± 0.3 × 10^8^ CFU ml^–1^) and WT^C^ (2.2 ± 0.3 × 10^8^/1.9 ± 0.4 × 10^8^/2.0 ± 0.2 × 10^8^ CFU ml^–1^), indicating efficient complementation of *∆spoVID* at *amyE* (Table 1). In contrast, the *spoVID^∆^^E^*, *spoVID^L125A^* and *spoVID^I127A^* mutations, but not *spoVID^L131A^* or *spoVID^I133A^*, specifically reduced the titer of lysozyme‐resistant spores, consistent with a defect in spore coat assembly (Table 1). The reduction in spore lysozyme resistance for the *spoVID^∆^^E^* (1.8 ± 0.7 × 10^6^ CFU ml^–1^), *spoVID^L125A^* (3.2 ± 1.8 × 10^6^ CFU ml^–1^) and *spoVID^I127A^* (1.0 ± 0.7 × 10^7^ CFU ml^–1^) strains, however, was greater than that caused by the *safA *deletion (7.4 ± 2.5 × 10^7^ CFU ml^–1^), and also by Δ*spoVID* (3.6 ± 1.0 × 10^7^ CFU ml^–1^) or disruption of *cotE *(4.5 ± 1.9 × 10^7^ CFU ml^–1^), all of which have severe coat assembly defects (Table 1). Thus, the mislocalization of SafA and/or CotE in *spoVID^∆^^E^*, *spoVID^L125A^* and *spoVID^I127A^* has a more pronounced effect on lysozyme resistance than their absence, or the absence of SpoVID.

The lysozyme susceptibility phenotype of *spoVID^∆^^E^*, *spoVID^L125A^* and *spoVID^I127A^* mutants suggested that spores of these mutants would have a drastically altered profile of extractable coat proteins. To test this, spores were purified through density gradients of metrizoic acid, decoated and the collection of extractable coat proteins examined by SDS‐PAGE and Coomassie staining (Fig. 5C). At least five bands (A and C and E to J, red arrows on Fig. 5C) showed increased extractability from spores of the *spoVID^∆^^E^*, *spoVID^L125A^* and *spoVID^I127A^* mutants but not from *spoVID^T126A^*, *spoVID^L131A^* or *spoVID^I133A^* spores, relative to the WT. Bands with the same mobility (A and C and E to J) are also more extractable from spores of a Δ*safA *mutant. Three species (D, K and L, blue arrows on Fig. 5C) appeared less extractable from *spoVID^∆^^E^*, *spoVID^L125A^* and *spoVID^I127A^* spores as compared with WT or *∆safA* spores. Species B and a group of bands below C and D (green arrows on Fig. 5C) were more extractable from Δ*safA* than from *spoVID^∆^^E^*, *spoVID^L125A^* and *spoVID^I127A^* spores. Thus, the coat protein extraction profiles of the *spoVID^∆^^E^*, *spoVID^L125A^* and *spoVID^I127A^* mutants, which showed a reduction in lysozyme resistance, resembled more closely that of a Δ*safA *mutant, whereas the profiles of the *spoVID^T126A^*, *spoVID^L131A^* or *spoVID^I133A^* mutants, which showed no reduction in lysozyme resistance, were more similar to that of WT spores (Fig. 5C and Table 1). Differences in the coat protein profiles, relative to the WT that are not shared by L125A, I127A or *∆safA* spores may be related to the gain function nature of the *spoVID^L125A^* and *spoVID^I127A^* alleles (see above). In any event, that the *spoVID^L125A^* and *spoVID^I127A^* mutations caused changes close to those observed with the *∆safA* allele is in line with the idea that the L125A and I127A substitutions prevent proper assembly of SafA.

### Region E substitutions L125A, I127A and I133A phenocopy a *spoVID *deletion

**Figure 6:**
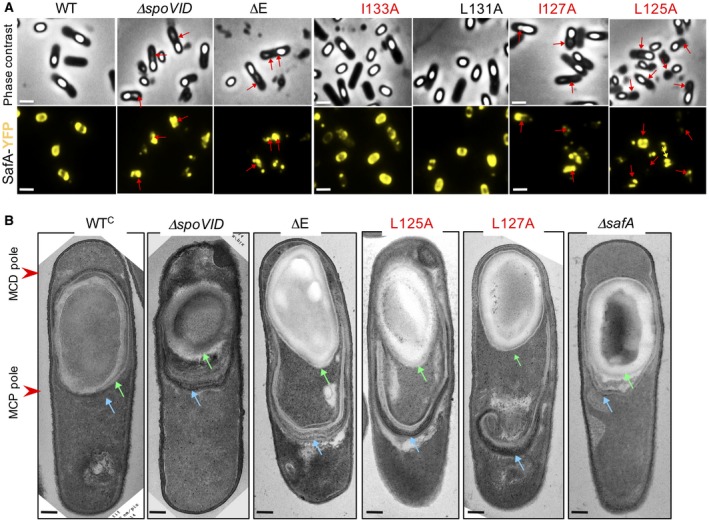
Deletion of E and the L125A and I127A substitutions cause a strong coat mislocalization phenotype. A. The indicated strains, expressing a *safA‐yfp* fusion were incubated in SM medium and samples collected 8 h after the onset of sporulation. The samples were examined by phase contrast and fluorescence microscopy. The red arrows point to misassembled coat material bound to one of the spore poles. Scale bar, 1 µm. B. Transmission electron microscopy analysis of *spoVID *mutant sporangia. Samples were collected from cultures 8 after the onset of sporulation in SM medium and processed for electron microscopy. WT^C^ denotes a *spoVID* in‐frame deletion mutant complemented with the WT allele at *amyE*. Green arrows point to the inner coat or inner coat‐like material and the blue arrows to the outer coat or outer coat‐like material. The position of the MCP and MCD spore poles is indicated by the red arrowhs. Scale bar, 0.5 µm.

To more precisely define the phenotype caused by region E mutations, we first examined sporulating cells of the various mutants additionally expressing a *safA‐yfp* fusion, 18 h after the onset of sporulation, a time when most of the sporangia have produced phase bright spores. Using phase contrast and fluorescence microscopy we found strong accumulation of partially refractile material near the MCP spore pole in strains lacking region E or producing SpoVID^L125A^ or SpoVID^I127A^, but not in those producing SpoVID^L131A^ or SpoVID^I133A^ (Fig. 6A, red arrows). Occasionally, in the SpoVID^L125A^‐producing strain, large masses of material were also seen attached to free spores (Fig. 6A, red arrows). Since SafA‐YFP often co‐localized with this material, in either sporangia or free spores, it most likely corresponds to misassembled coat proteins (Fig 6A; compare the phase contrast with the fluorescence images). Moreover, this phenotype is also seen for *spoVID *mutants (Beall *et al*., 1993) (Fig. 6A). In contrast, misassembly of the coat in a Δ*safA *mutant is not detected by phase contrast microscopy (but see below). *spoVID^∆^^E^*, *spoVID^L125A^* and *spoVID^I127A^* mutants have more severe assembly defect than *spoVID^131A^*, *spoVID^133A^* or Δ*safA *mutants, suggesting a gain of function effect (above). The phenotype revealed by the phase contrast/fluoresce microscopy analysis of the mutants hinted at the origin of the gain of function effect of the *spoVID^∆^^E^*, *spoVID^L125A^* and *spoVID^I127A^* alleles, i.e. the massive accumulation of coat material at the MCP spore pole. The association of misassembled coat material with spores during their purification may explain why the collection of extractable coat proteins does not correlate with the severity of the lysozyme susceptibility phenotype among the various mutants.

We examined sporangia of these mutants by transmission electron microscopy (TEM) of samples collected at hour 8 of sporulation. While in the WT or the WT^C^ strain, the coat (blue arrow in Fig. 6B) formed close to the edge of the spore cortex (green arrow), in *spoVID^∆^^E^*, *spoVID^L125A^* and *spoVID^I127A^* sporangia, coat material projected from the MCP spore pole into the mother cell cytoplasm (Fig. 6B). Reminescent of a *spoVID *mutant (Beall *et al*., 1993), this material maintained the general organization of the coat formed around WT spores in that it showed more lamellar internal sheet and more electrondense outer sheet (Fig. 6B). Δ*safA *sporangia showed thin projections of material into the mother cell cytoplasm but these showed no apparent organization into inner coat/outer coat‐like material (Fig. 6B). These differences are consistent with the role of SafA in inner coat assembly and with the previous characterization of the mutant (Takamatsu *et al*., 1999; Ozin *et al*., 2001a) and explain the failure to observe the misassembled coat by phase contrast microscopy.

Importantly, since the L125A substitution does not affect, and I127A only reduces slightly, the direct interaction of CotE with SpoVID but caused mislocalization of CotM‐, CotO‐ and CotE‐GFP ((de Francesco *et al*., 2012); this work), we infer that the mislocalization of SafA and of the SafA‐dependent proteins most likely serves as a strong attractor for the outer coat. This would explain the massive accumulation of coat material at the MCP pole in L125A, I127A and ΔE sporangia and the increased susceptibility to lysozyme of spores produced by these strains, relative to the Δ*safA *mutant.

### Encasement by SafA is largely independent from encasement by CotE

**Figure 7:**
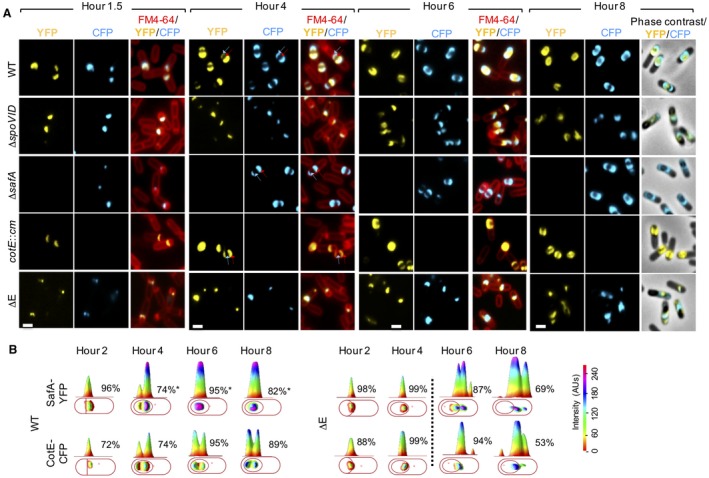
The simultaneous localization of SafA‐YFP and CotE‐CFP during spore encasement. A. Cultures of the indicated strains, producing SafA‐YFP and CotE‐CFP were incubated in SM medium, and samples collected at hours1.5, 4, 6 and 8 after the onset of sporulation. The cells were stained with the membrane dye FM‐4‐64 and analyzed by fluorescence microscopy for the localization of SafA‐YFP and CotE‐CFP. YFP and CFP signals are superimposed on the FM4‐64 signal (for hours 1.5, 4 and 6) or o phase contrast images (hour 8). Scale bar, 1 µm. B. One representative cell of the prevalent pattern was chosed to show the distribution of the fluorescence signal in three dimensional intensity graphs. The quantification of the various localization patterns is shown in Tables S4 and S5.

The proximity of the residues involved in the interaction of SafA and CotE with SpoVID, as well as the kinetics class II behavior of the two proteins prompted us to more precisely inspect the kinetics of assembly of SafA‐YFP and CotE‐CFP relative to each other. The SafA‐YFP fusion (at *amyE*) and the CotE‐CFP fusion (integrated by a single cross‐over at the 3′‐end of the *cotE* locus) were introduced into the same WT strain, as well as in the mutants missing *safA*, *cotE*, *spoVID* or expressing the *spoVID^∆^^E^* *a*llele from the native *spoVID *locus (above). The localization of SafA‐YFP and CotE‐CFP was then monitored throughout sporulation in the same cells. We scored the percentage of sporangia with no cap, one cap or two caps of CotE‐CFP in sporangia, where SafA‐YFP was found as either one or two caps. At hour 2 in the WT, 72% of sporangia with 1 cap of SafA‐YFP also showed one cap of CotE‐CFP the remaining showing no CFP signal. At hour 4 of sporulation, 25% of the sporangia with one cap of SafA‐YFP showed a cap of CotE‐CFP and two caps were found for both SafA‐YFP and CotE‐CFP in 74% of the sporangia (Figs. 7; Table S5). Finally, at hour 6, 95% of the sporangia with two caps or nearly complete rings of SafA‐YFP also showed two caps of CotE‐CFP (we note that CotE‐CFP formed two nearly connected caps in all sporangia scored; the absence of a complete ring may be due to impaired functionality of the fusion protein). This suggests that SafA localizes earlier and expands faster at the MCP pole. For both SafA‐YFP and CotE‐CFP, the fluorescence signal at the MCD pole was often weaker than that at the MCP pole (Figs. 7; Table S4). The weaker signal at the MCD pole most likely represents an early stage in expansion at this cap but we found no significant difference between the two fusion proteins. In Δ*spoVID* or *spoVID^∆^^E^* cells some cells with SafA‐YFP and CotE‐CFP spread throughout the mother cell cytoplasm were observed at late times of sporulation, and in these cells the YFP and CFP signals overlap (Figs. 7; Table S4). These phenotypes are in line with the idea that the mislocalized inner coat serves as an attractor for CotE and the outer coat.

Finally, we examined the localization of SafA‐YFP in a *cotE* insertional mutant and conversely, the localization of CotE‐CFP in a Δ*safA* mutant. We found no difference in the localization kinetics of either protein in the mutants (Fig. 7; Table S5) and the difference in the signal intensity between the two poles, as described above, was maintained for SafA and for CotE. Thus, we found no evidence suggesting that SafA and CotE compete for SpoVID during encasement.

## Discussion

**Figure 8:**
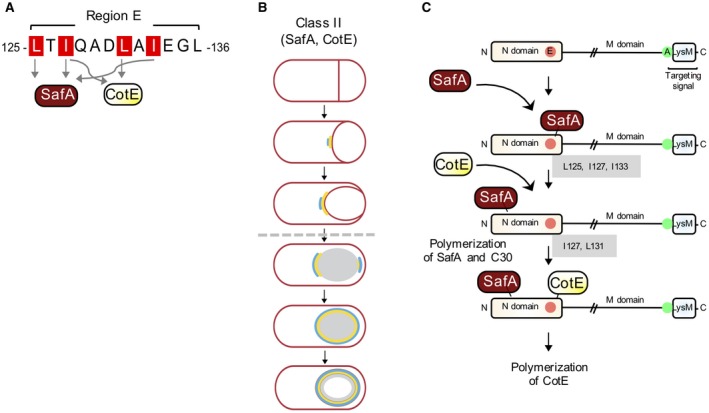
Model for the interaction of SafA and CotE with SpoVID during encasement. A. SafA and CotE interact directly with region E of SpoVID. L125 and I133 are specifically involved in the interaction of SafA with SpoVID; L125A blocks encasement while I133A delays encasement. I127A impairs both the interaction of SafA and CotE with SpoVID, while L131 specifically affects the interaction with CotE. B. both SafA and CotE are kinetics class II proteins that form a cap at the MCP pole at the onset of engulfment. SafA localizes earlier than CotE at this pole, and the cap it forms expands faster than the cap formed by CotE. Following engulfment completion (broken arrow) SafA and CotE localize at the MCD spore pole, but CotE localizes earlier or faster at this site. Both proteins completely encase the spore concomitantly. C. a two‐step model for the interaction of SafA with SpoVID, via E. SafA initially binds to E using L125, I127 and I133. This interaction allows SafA to recognize a diferent region in SpoVID, possibly within its N domain, required for encasement, liberating E. This step may promote polymerization of SafA and C30, which binds to itself and to the C30 region in SafA^FL^. CotE may then interact with E, using I127 and L131. Binding of CotE to E may also stimulate or otherwise control polymerization of CotE. Successive cycles of SafA and CotE binding to E may occur during encasement, minimizing competion between the two proteins for E in SpoVID. SpoVID in turn, would act essentially as a non‐competitive hub.

Region E of the N‐terminal domain of SpoVID is essential for forespore encasement by all layers of the coat. Previously, two residues in E, I127 and I131, were shown to be important for interaction with CotE and forespore encasement by CotE and CotE‐dependent proteins (Fig. 1C). We have now expanded these studies and showed that residues L125, I127 and I133 in E are necessary for interaction with SafA and required for efficient forespore encasement by SafA and the SafA‐dependent proteins that form the inner coat layer of the spore (Fig. 8A). Ala substitutions for each of these three residues prevent the interaction with SafA, indicating that encasement relies on a direct interaction between SafA and region E of SpoVID. L125A, I127A and I133A all delay formation of a SafA‐YFP cap at the MCP spore pole, but only L125A and L127A also prevent formation of the MCD cap, implying that L125 and I127 have a more important role than I133 in encasement by SafA.

Importantly, the L125A and I127A substitutions cause, over time, a strong accumulation of coat material at the MCP spore pole (Fig. 6). In TEM images, this material appears to be composed of juxtaposed layers with morphological features reminiscent of the inner and outer coat as seen around WT mature spores or in the mother cell cytoplasm in *∆spoVID* or *spoVID^∆^^E^* mutants (Beall *et al*., 1993; this work). This would suggest that the inner coat material that accumulates at the MCP pole acts as a strong attractor for CotE and the outer coat. Thus, CotE and outer coat material appear to have more affinity for a misassembled inner coat than available binding sites in SpoVID. I127A may act in a similar way, although, in this case, the substitution also reduces the interaction between CotE and SpoVID. The trapping of most of the coat at the MCP spore pole exposes cortex peptidoglycan explaining the higher degree of susceptibility to lysozyme of the L125A and I127A mutant spores compared to the Δs*afA* spores, which remain surrounded by the outer coat (Figs. 3 and 6; Table 1). The impaired interaction between SafA and SpoVID does not result in mislocalization of SpoVID, as SpoVID^∆E^ and the forms of the protein with the various single amino acid substitutions all localize normally in a Δ*safA* mutant (Wang *et al*., 2009). Moreover, SpoVID, as well as all the altered forms of the protein tested in this work, are found in the spore ‘lysozyme fraction’ (Fernandes *et al.*, 2018), which presumably corresponds to the cortex or cortex/inner coat interface (Fig. 5). The localization of SpoVID is a consequence of an interaction of its C‐terminal region A with the basal layer protein SpoIVA and on a LysM domain, which may mediate an interaction with peptidoglycan in the intermembrane space and/or with peptidoglycan precursors.

Thus, while the recruitment of inner and outer coat proteins results from largely independent interactions with SafA and CotE, respectively, a strong connection exists between these two coat sub‐structures. We have recently proposed the existence of such a strong interaction between the inner and outer coat based on the phenotype arising from production of C30 independently of full‐length SafA (Fernandes *et al.*, 2018). C30 lacks the localization signals located at the N‐terminal end of the full‐length protein, which include a short region, termed A, known to mediate a direct interaction with SpoVID (Fig. 1D). Thus, C30 cannot interact with SpoVID and does not localize unless put in the presence of SafA^FL^, with which it interacts (Ozin *et al*., 2001b). Significantly, production of C30 in the absence of SafA^FL^ also causes strong accumulation of partially structured inner and outer coat material at the MCP pole and throughout the mother cell cytoplasm, i.e. blocking encasement and mimicking the phenotype of the L125A and I127A mutants (Fernandes *et al.*, 2018). C30, alone or in a complex with SafA^FL^, is clearly the domain of SafA responsible for the recruitment of the inner coat proteins to the spore surface, and for the accumulation of the inner and outer coat at the MCP spore pole when encasement by SafA is blocked ((Fernandes *et al.*, 2018); this work).

Several forms of SafA are present in both the spore cortex and coat ((Fernandes *et al.*, 2018); this work, Fig. 5A). The interaction of SafA with SpoVID is important for distributing the protein among the different layers of the spore, which is likely to be established during encasement. The final localization of SafA in the cortex or the cortex/inner coat interface as determined by immunogold labeling (Ozin *et al*., 2000) and fractionation studies ((Fernandes *et al.*, 2018); Fig. 5A) is consistent with the presence of a LysM domain in the protein and it seems likely that this domain contributes to the assembly of SafA. If so, the LysM domain may be required in addition to SpoVID for encasement by SafA‐YFP. The contribution of this domain to the assembly of SafA, however, has not yet been tested. It is also unclear how SafA would reach the cortex region across the outer forespore membrane since, as is the case for SpoVID, the proteins lack any known secretion signals. In any event, since deletion of E and the L125A, I127A and I133A substitutions strongly reduce the extractability of SafA and C30 from the cortex (Fig. 5A and B), the final localization of the protein is established, at least in part, via its interaction with SpoVID during encasement.

Our study updates the model describing forespore encasement by the coat proteins (Fig. 8). SpoVID localizes at the forespore surface via an interaction of its C‐terminal localization domain with SpoIVA. A second interaction with SpoIVA is likely to occur, presumably involving the N‐terminal domain of SpoVID, as encasement by SpoIVA requires SpoVID (de Francesco *et al*., 2012; McKenney and Eichenberger, 2012). Whether SpoVID interacts directly with SpoIVA via region E has not yet been tested (de Francesco *et al*., 2012). At the onset of engulfment, SpoVID interacts with SafA, with L125, I127 and I133 mediating the interaction, and with CotE, specifically via I131 but with the participation of I127 (Fig. 8A). Thus, the residues important for the interaction of CotE and SafA with E partially overlap.

SpoVID acts as a higher‐level organizing hub, connecting the lower level semi‐autonomous basal layer, inner coat, outer coat and, perhaps, crust assembly modules (Jin *et al*., 2007; Agarwal *et al*., 2010; Dasgupta *et al*., 2011; Goel and Wilkins, 2012). Higher‐level organizing connectors may have only one or two binding surfaces that are used by multiple partners sequentially and/or at different cellular locations, since the interactions may be mutually exclusive (Jin *et al*., 2007; Dasgupta *et al*., 2011; Goel and Wilkins, 2012). SpoVID seems to fit this description. It is not known whether SafA and CotE bind simultaneously or mutually exclusively to SpoVID. It has been suggested previously that SafA, assumed to be a kinetics class I protein, is released from SpoVID, allowing binding of and encasement by CotE, a kinetics class II protein (de Francesco *et al*., 2012). Thus, the interactions of SafA and CotE with SpoVID would be sequential. SafA would track the engulfing membranes and would complete encasement before CotE, which encases the forespore from the MCD cap formed after engulfment completion (McKenney and Eichenberger, 2012). The different kinetics of assembly by SafA and CotE would thus minimize the problem of simultaneous binding of SafA and CotE to SpoVID. In our study, however, in which a functional fusion was used, SafA‐YFP (and the SafA‐dependent YaaH‐GFP fusion) behaved as kinetics class II proteins (Fig. 8B). SafA and CotE may thus compete for binding to E and the interaction of either protein would be mainly controlled by their local concentration. While the kinetics of encasement by SafA‐YFP was studied for the first time herein, the discrepancy in assignment of YaaH‐GFP to a kinetics class between this study and that of de Franscesco and colleagues (de Francesco *et al*., 2012) may result from differences in culturing conditions as the strain background and the YaaH‐GFP fusion are the same (de Francesco *et al*., 2012). In any case, two observations suggest that competition between SafA and CotE for E may be negligible. Firstly, the interaction of SafA or CotE with region E seems to allow a second interaction of the two proteins with another, yet unidentified, binding interface in SpoVID (Fig. 8B). It is possible that the interaction of SafA or CotE with region E alters their conformation, allowing the proposed secondary interaction. The PYYH motif in the C30 region, identified in a phage display screen for SpoVID interactors, may contact SpoVID but this interaction does not seem to be required for encasement (Ozin *et al*., 2000) (Fig. 1D). Furthermore, studies using molecular force microscopy have shown that CotE interacts with regions of SpoVID outside the N‐terminal morphogenetic domain (Qiao *et al*., 2012; 2013). In this model, the interaction of SafA or CotE with E would be transient, and region E would not be permanently occupied (Fig. 8C). It has been suggested previously that polymerization of SafA releases it from SpoVID, allowing an interaction with CotE and the polymerization of the latter (de Francesco *et al*., 2012). If, as we suggest, the interaction of SafA and CotE with E is transient, then polymerization of SafA and CotE, nucleated by their interaction with E, can occur through cycles of binding to E and their transfer to other regions of SpoVID and to nascent polymers (Fig. 8D). Secondly, assembly of SafA and CotE is partially segregated in time as CotE appears to localize slightly later than SafA at the MCP pole even when SafA is absent. As discussed above, the localization of SafA may also be influenced, through its LysM domain, by the pattern of peptidoglycan synthesis during engulfment.

In total, our results suggest that SpoVID functions as a non‐competitive hub (Jin *et al*., 2007; Dasgupta *et al*., 2011; Goel and Wilkins, 2012). The apparent binding promiscuity of SpoVID, in that SafA, CotE and possibly also SpoIVA, may all bind directly (even though transiently, as this study now suggests) to E, brings the question of what are the structural features of this region that allows binding by different proteins with sufficient specificity and affinity, and what are the features that define the recognition regions in its partners. An answer to these questions will be essential to understand the role of SpoVID in orchestrating proper assembly of the inner and outer spore coat layers.

## Experimental procedures

### Bacterial strains, media and general methods

All *B. subtilis* strains used in this study are congenic derivatives of the standard Spo^+^ laboratory strain PY79 and are listed in Table S6. Strain DH5α of *Escherichia coli *was used for the construction of all the plasmids used in this work, described in detail in the Supporting information. The construction of all plasmids used herein is described in detail in the Supporting information ; oligonucleotides are listed in Table S7 and plasmids are listed in Table S8. Routine growth of *E. coli* and *B. subtilis* strains were done in Luria‐Bertani medium at 37°C, supplemented with appropriate antibiotics when needed. Sporulation was induced by growth and resuspension into Sterlini–Mandelstam medium (SM) (Sterlini and Mandelstam, 1969).

### GST pulldown assays

For the overproduction of native glutathione *S*‐transferase (GST) and GST fused to SpoVID (either the wild‐type protein or mutant forms bearing a deletion of region E or single alanine substitutions in region E residues), the derivatives of *E. coli *CC118 (DE3)/pLysS listed in Table S6 were used. For the overproduction of SafA, the *E. coli* BL21 (DE3) derivative AH5236 was used. Cultures of 10 ml (for GST and SafA) or 50 ml (for the GST‐SpoVID variants) were grown to an optical density at 600 nm of 0.6 and induced with 1 mM IPTG for 3 h (for GST and GST‐SpoVID variants) or for 30 min (for SafA). Cells were harvested by centrifugation (at 4°C for 10 min at 7500 × *g*) and resuspended in 1 ml of cold buffer. VPEX‐100 buffer (100 mM NaCl, 10 mM Tris pH 8.0, 1 mM EDTA, 1 mM 2‐mercaptoethanol, 0.1% Triton X‐100, 1 mM phenylmethylsulfonyl fluoride, 10% glycerol (Ozin *et al*., 2001b)) was used to resuspend the cells producing GST or the various GST‐SpoVID proteins, while buffer I (PBS containing 0.1% Tween 20 + 10% glycerol) was used for the resuspension of SafA producing cells, both supplemented with 1 mM phenylmethylsulfonyl fluoride and Complete Mini EDTA‐free protease inhibitor cocktail (Roche). Cell lysis was performed in a French pressure cell (18,000 lb/in2) or in a QSonica CL5 sonicator (Misonix) (amplitude of 50 for 30 s, repeated 4 times with 10 s intervals). Lysates were cleared by centrifugation (at 4°C for 20 min at 10000 × *g*). GST and GST‐SpoVID amounts in the soluble fractions were adjusted with VPEX‐100 (above) and a SafA cleared lysate was diluted 1:500 in buffer I. Pull‐down assays were performed as previously described (de Francesco *et al*., 2012), except that GST and GST‐SpoVID variants were used as baits and SafA was used as prey. As a negative control, a BL21 (DE3) cleared lysate was also incubated with GST‐SpoVID. Bead fractions were resuspended in SDS protein loading buffer (10% glycerol, 4% SDS, 10% 2‐mercaptoethanol, 1 mM DTT, 250 mM Tris pH 6.8, 0.05% bromophenol blue), boiled for 5 min, and proteins resolved on 12.5% SDS‐PAGE gels. Gels were transferred to nitrocellulose membranes for immunoblot analysis with anti‐SafA antibodies, and the membranes were finally stained with Ponceau S to control for retention of GST or the GST‐SpoVID variants.

### Analysis of the spore coat proteins

Spores were purified from 24 h cultures after resuspension in SM medium using a two‐step gradient of Gastrografin (Bayer Schering Pharma) (Seyler *et al*., 1997; Henriques and Moran, 2000). Total coat proteins were extracted by boiling the spores (equivalent to an OD_580 nm_ of 2) for 8 min with extraction buffer (10% glycerol, 4% SDS, 10% β‐mercaptoethanol, 1 mM DTT, 250 mM Tris pH 6.8, 0.05% bromophenol blue) and resolved on 15% SDS‐PAGE gels (Costa *et al*., 2006). The gels were stained with Coomassie brilliant blue R‐250. For decoating assays, spores (2 OD units at 580 nm) were boiled for 5 min in extraction buffer without bromophenol blue and the samples were then centrifuged for 2 min at 16200 × *g*. The supernatant, corresponding to the soluble coat fraction, was removed and placed in a new tube and bromophenol blue was added (final concentration of 0.05%) before analysis by 15% SDS‐PAGE. The sediment, corresponding to decoated spores, was washed twice with TBST (50 mM Tris, 150 mM NaCl, pH 8.0, h 0.1% Tween 20) and divided into two equal volume samples that were incubated at 37°C for 2 h in 50 mM Tris buffer (pH 8.0) with or without lysozyme (2 mg ml^‐1^) (Fernandes *et al.*, 2018). Then, the samples were incubated 5 min at 100°C with SDS protein loading buffer and resolved, along with the coat fraction (above), on 15% SDS‐PAGE gels. The gels were transferred to nitrocellulose membranes for immunoblot analysis using anti‐SafA and anti‐SpoVID antibodies (Ozin *et al*., 2000). As a control for the decoating, the membranes were incubated in a stripping buffer (50 mM Tris, pH 6.8, 2% SDS, 100 mM β‐mercaptoethanol) and reprobed with anti‐CotA antibodies (Martins *et al*., 2002).

### Immunoblot analysis

Immunoblot analysis was conducted using the SuperSignal West Pico Chemiluminiscent Substrate (Thermo Scientific) according to the manufacturer´s instructions and using 5% low fat powder milk in PBS‐0.1% Tween‐20 (0.001%) as the blocking agent. Antibodies were used at the following dilutions: anti‐SafA 1:15000 (Ozin *et al*., 2000), anti‐CotA (Martins *et al*., 2002), 1:1000, anti‐σ^A^, 1:1000 (Fujita and Losick, 2002). Secondary horseradish peroxidase‐conjugated antibody (Sigma) was used at a concentration of 1:5000.

### Peptide‐binding assays

Three synthetic N‐terminal biotinylated peptides with sequences derived from E were used in these experiments: the peptide corresponding to the WT sequence plus 3 residues upstream and one downstream (peptide pE^WT^: SRILTIQADLAIEGLLD), and a variant with an alanine substitutions of residue L125 in region E, termed pE^L125A^. Peptides had purities of > 73.121%, > 92.361% and > 83.514% (ThermoFisher Scientific), respectively, and were diluted in PBS‐0.1%‐Tween 20. Overproduction of GST, GST‐SpoVID variants or SafA and preparation of the lysates was as described above. Pull‐down assays were performed as described above (section on GST pull‐down assays), except that peptides were added to 1 ml of the diluted lysates containing SafA or the GST‐SpoVID variants, depending on the binding assay, for the final concentrations of 1, 10 or 100 µM and incubated (at 4°C with agitation for 1 h) before addition to the beads fraction. SafA was detected by imunoblotting.

### Fluorescence microscopy and image analysis

The cells in 1 ml samples of SM cultures were collected by centrifugation (4000 × *g*, 10 min, at 4°C) at the desired times after the onset of sporulation, washed with 1 ml of phosphate‐buffered saline (PBS), and ressuspended in 0.1 ml of PBS supplemented with 1 µl of a 2 mg ml^–1^ solution of the membrane dye FM4‐64 (Molecular Probes, Invitrogen). Fluorescence microscopy was performed with a Leica DM6000B or a Nikon 90i microscope as previously described (Serrano *et al*., 2015). In the Leica microscope, fluorescent signals were visualized with a phase contrast objective Uplan F1 100× and captured with a CCD camera Andor Ixon^EM^ (Andor Technologies) using Metamorph version 5.8 (Universal Imaging), while in the Nikon microscope an objective Plan Fluor 1.3NA 100x (Nikon), a Roper 1K digital camera and the software NIS Elements AR 3.0 (Nikon) were used. Image analysis was conducted in Metamorph v5.8, NIS Elements AR 3.0 and ImageJ (https://rsbweb.nih.gov/ij/). For quantification of the subcellular localization patterns of each fluorescent fusion, at least 100 sporulating cells were randomly examined and scored. The 3D graphics representing the distribution of fluorescent intensity patterns in sporulating cells were performed for one representative cell using the ImageJ plugin Interactive 3D Surface Plot v2.33.

### Transmission electron microscopy

Samples from SM cultures were collected by centrifugation 8 h after the onset of sporulation, and the cells fixed and processed for thin sectioning TEM as described before (Fujita and Losick, 2002)
