# Supplementary figures and images for: SpoVID functions as a non‐competitive hub that connects the modules for assembly of the inner and outer spore coat layers in Bacillus subtilis
Source: Mol Microbiol. 2018 Oct 18;110(4):576–95. doi: 10.1111/mmi.14116 (PMC6282716; doi:10.1111/mmi.14116)

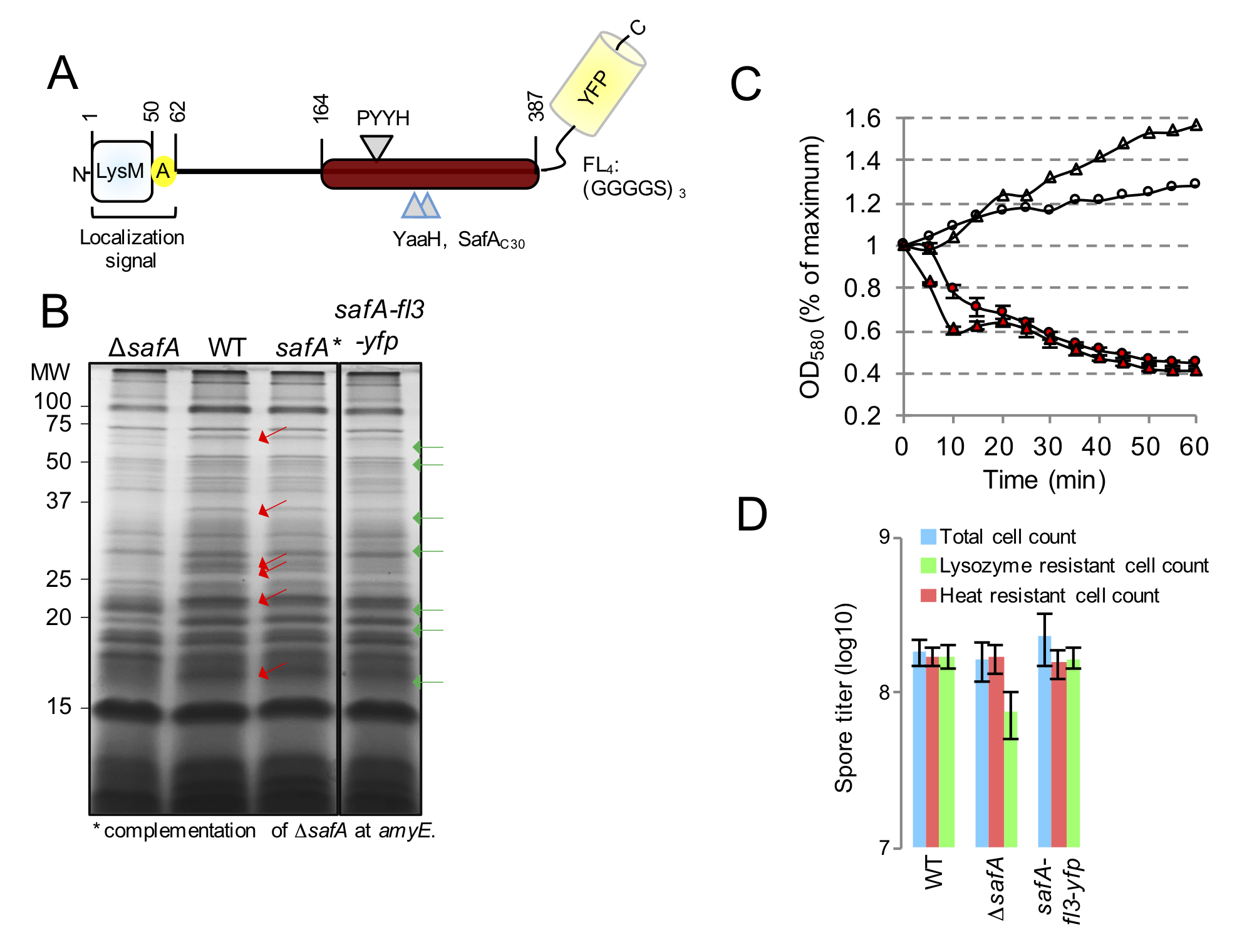

Supplement: Supplementary file 1 [file MMI-110-576-s001.tiff]

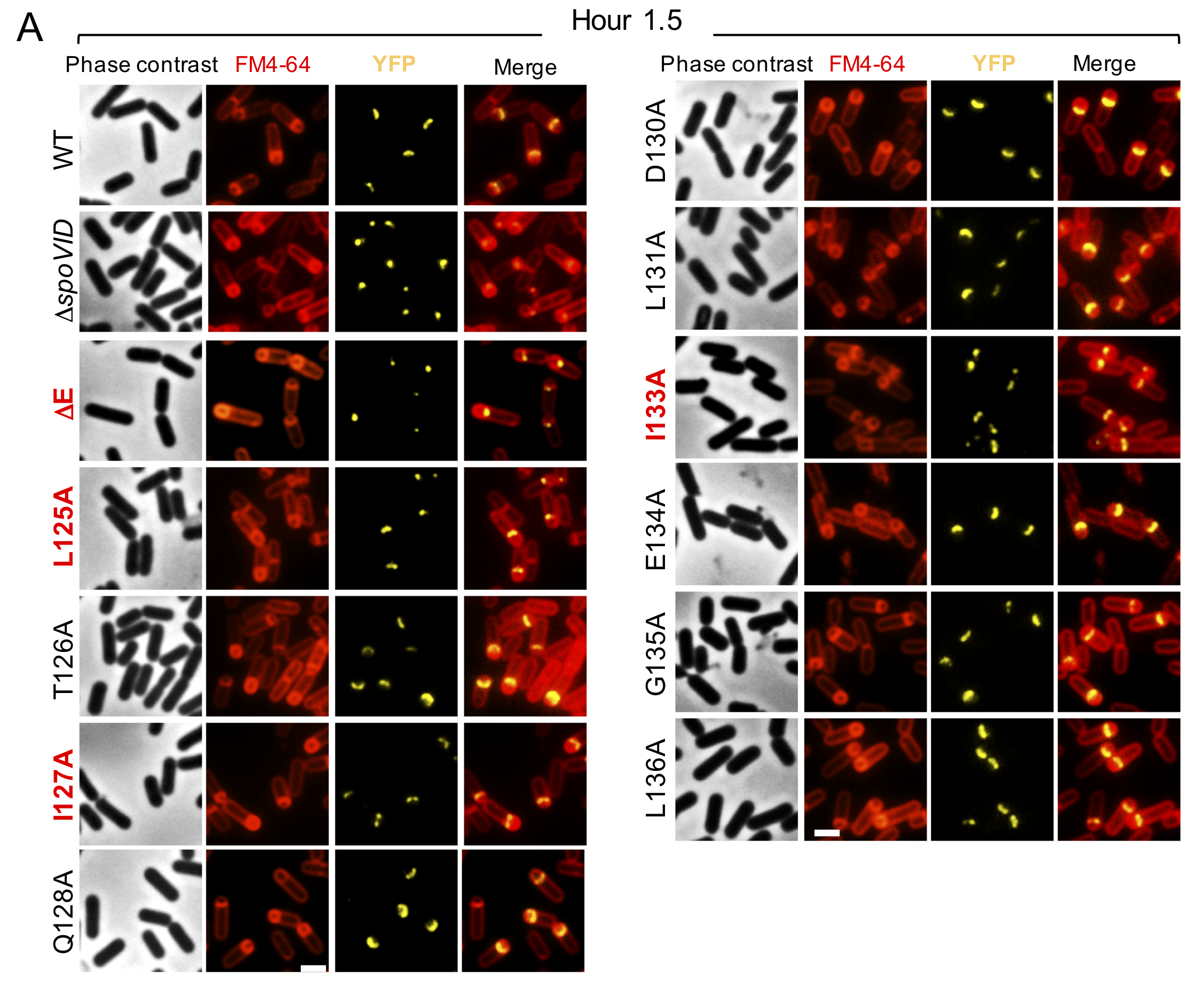

Supplement: Supplementary file 2 [file MMI-110-576-s002.tiff]

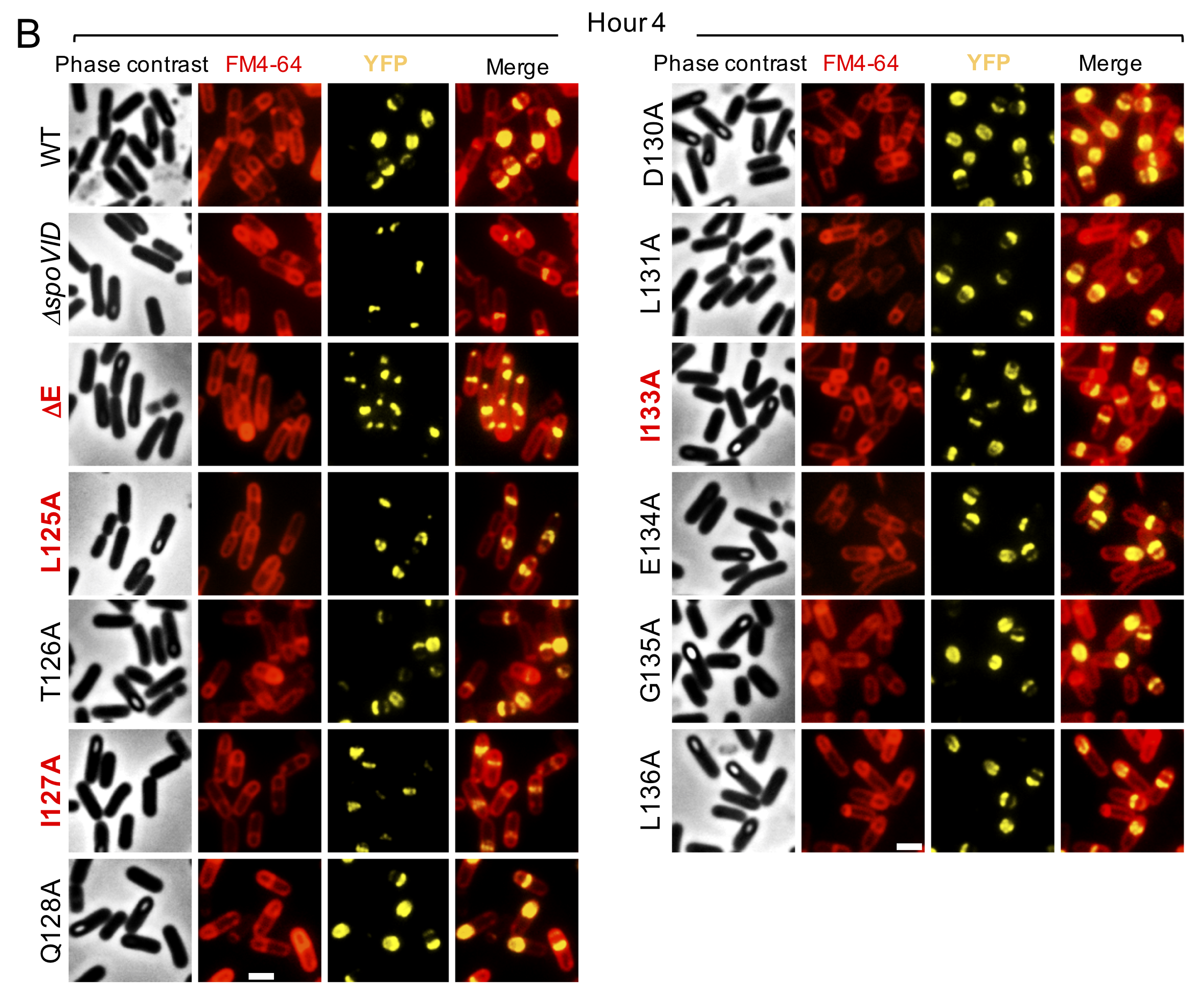

Supplement: Supplementary file 3 [file MMI-110-576-s003.tiff]

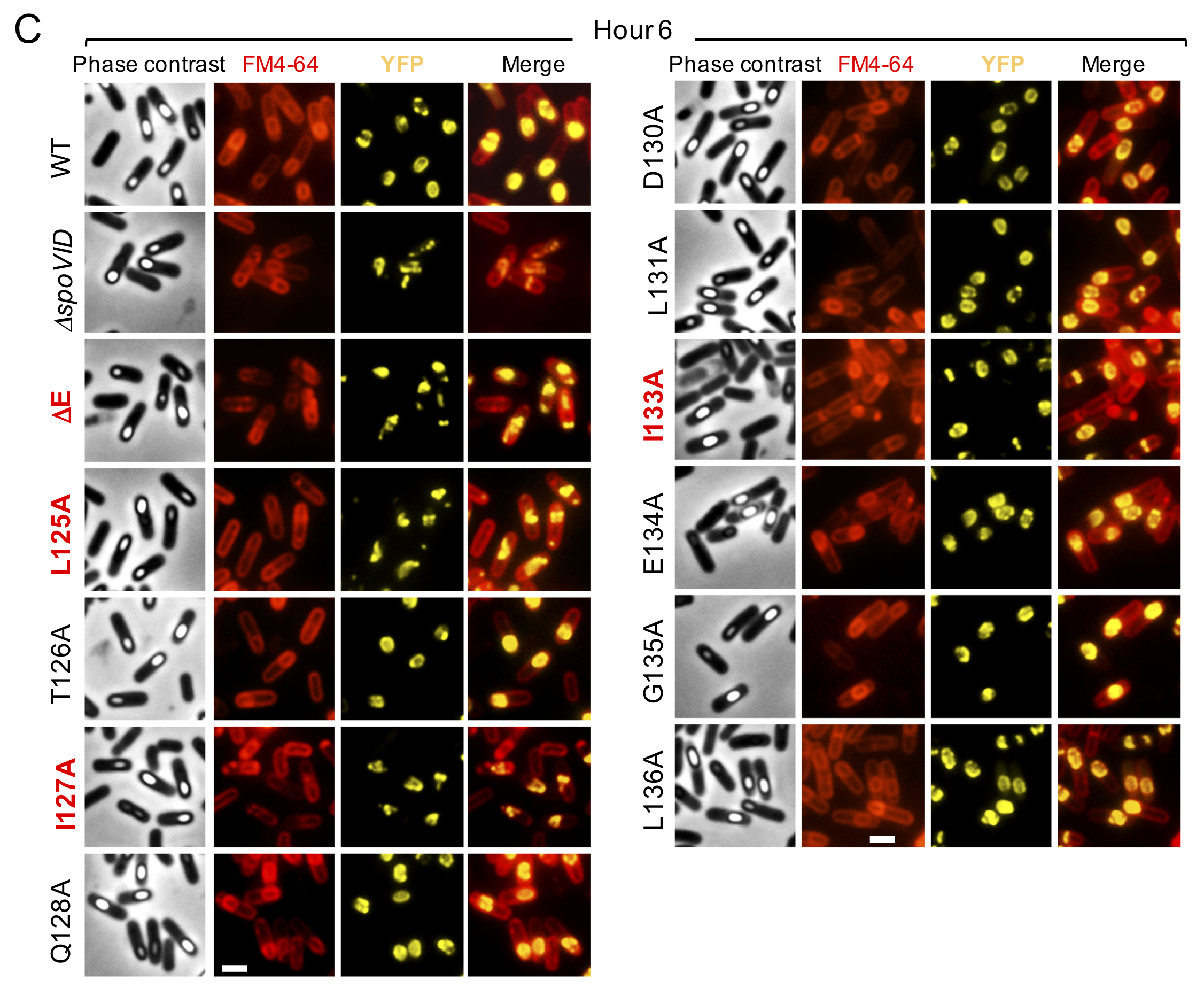

Supplement: Supplementary file 4 [file MMI-110-576-s004.tiff]

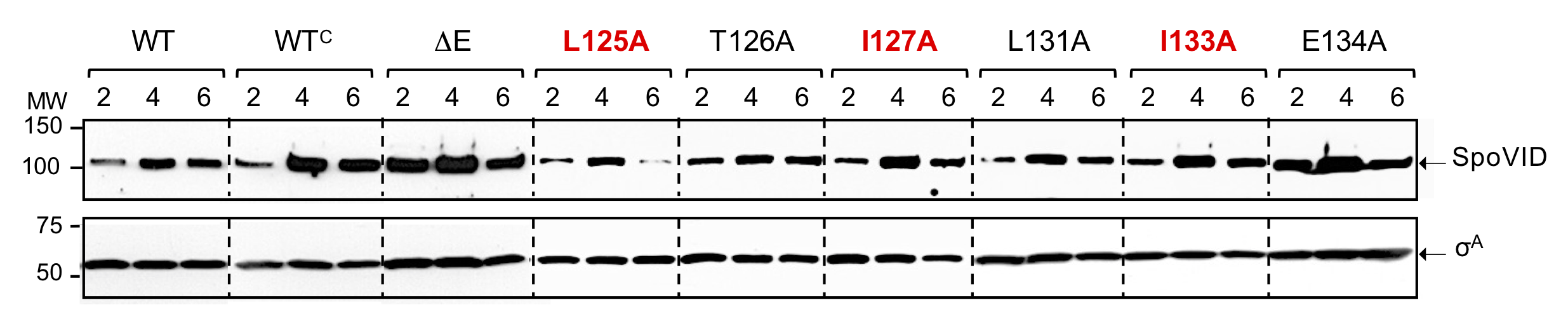

Supplement: Supplementary file 5 [file MMI-110-576-s005.tiff]

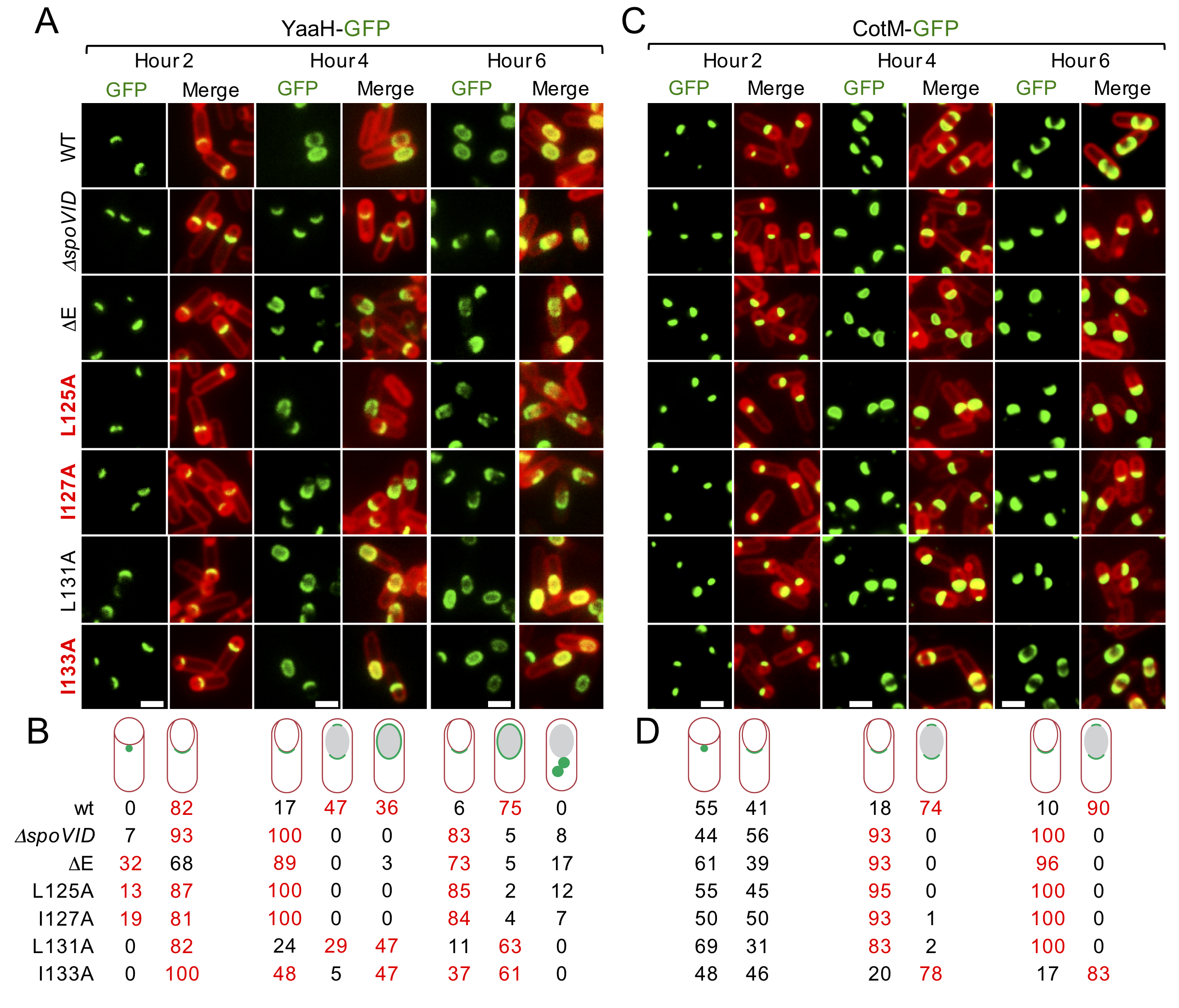

Supplement: Supplementary file 6 [file MMI-110-576-s006.tiff]

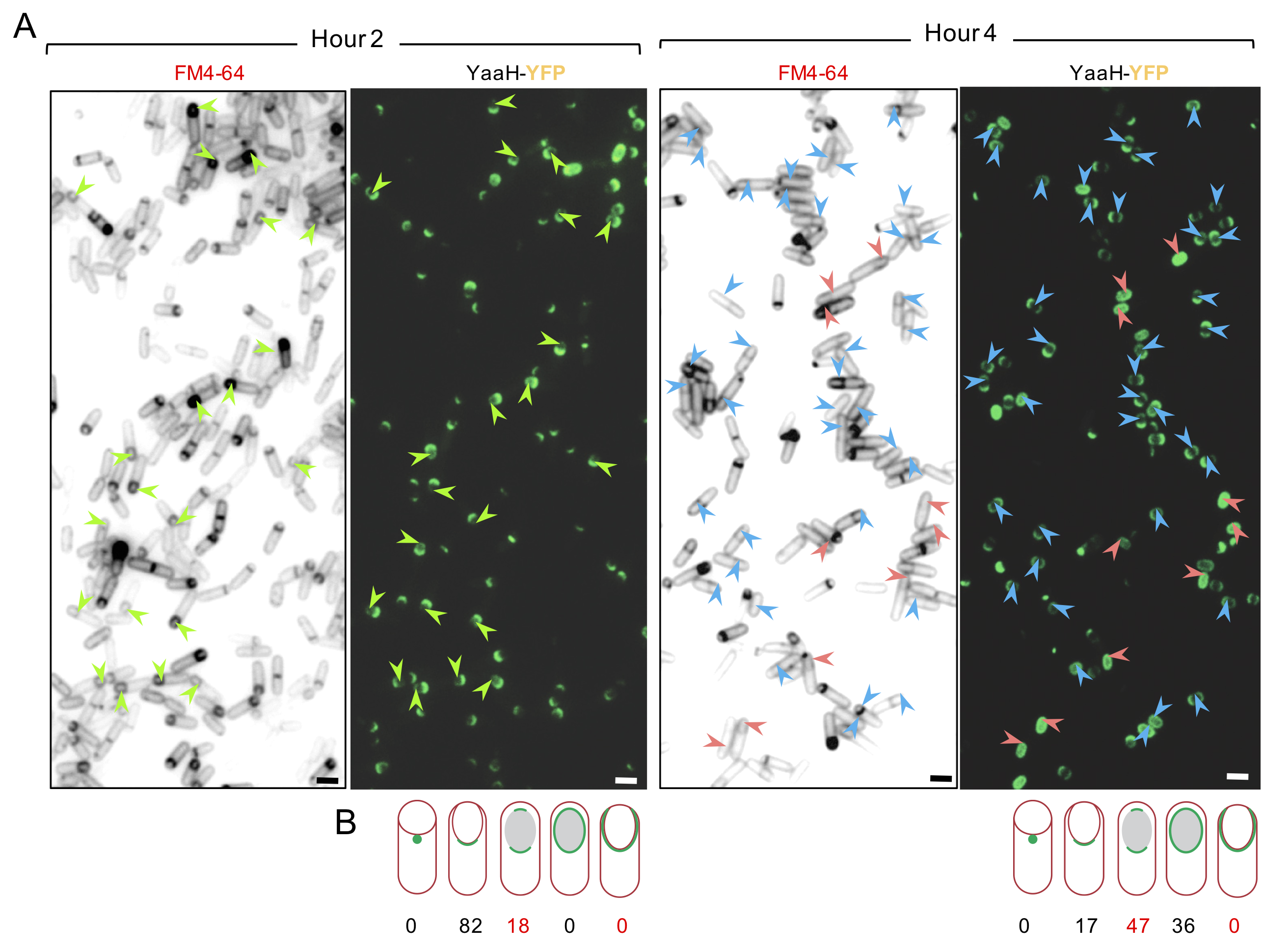

Supplement: Supplementary file 7 [file MMI-110-576-s007.tiff]

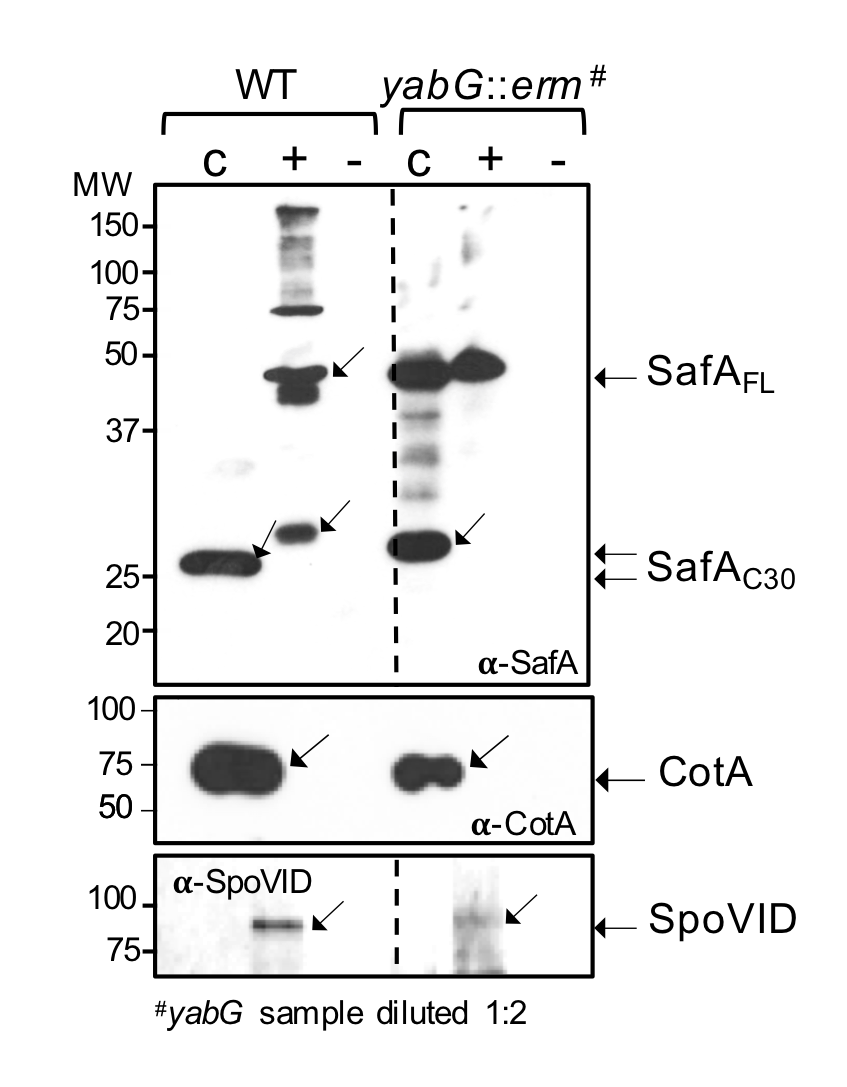

Supplement: Supplementary file 8 [file MMI-110-576-s008.tiff]
