# Supplementary material for: SpoVID functions as a non‐competitive hub that connects the modules for assembly of the inner and outer spore coat layers in Bacillus subtilis
Source: Mol Microbiol. 2018 Oct 18;110(4):576–95. doi: 10.1111/mmi.14116 (PMC6282716; doi:10.1111/mmi.14116)
Supplement: Supplementary file 9 [file MMI-110-576-s009.docx]

**Supporting Information**

**SpoVID functions as a non-competitive date hub that connects the modules for assembly of the inner and outer spore coat layers**

**in *Bacillus subtilis***

Filipa Nunes^1^, Catarina Fernandes^1^, Carolina Freitas^1^, Eleonora Marini^1^, Mónica Serrano^1^, Charles P. Moran Jr.^2^, Patrick Eichenberger^3^ and Adriano O. Henriques^1^

^1^Microbial Development Laboratory, Instituto de Tecnologia Química e Biológica, Universidade Nova de Lisboa, Oeiras, Portugal; ^2^Emory University School of Medicine, Depratment of Microbiology and Immunology, Atlanta GA 30322, USA; ^3^Department of Biology, New York University, New York, NY 10003, USA

**Supporting Material and Methods**

***spoVID* and *safA* in-frame deletion mutants**. An amplicon with the *spoVID* coding sequence with an in-frame deletion that eliminates residues 39 – 548 and the regions upstream and downstream *spoVID* was generated through splicing-by-overlay-extension (SOE)-PCR with primers VID-UP385Dir, VID-294Rev, VID-1846Dir and VID-2290Rev. The SOE-PCR product was cloned into pJET1.2/blunt (Thermoscientific), digested with BglII and inserted into the same restriction site of pMAD ([Arnaud *et al.*, 2004](#_ENREF_2)). This generated pFN87. SOE-PCR using primers safA-364D, safA+135R, safA+748D and safA+1250R creates a DNA fragment containing the *safA* coding region with an in-frame deletion that eliminates residues 46-249 and *safA* flanking regions. This fragment was digested with EcoRI and SalI and inserted in the same cloning sites of pMAD (Arnaud et al., 2004), producing pFN115. pFN87 and pFN115 were used to replace the *spoVID* or *safA* loci of the WT PY79 strain with the in-frame deletions, as described ([Arnaud *et al.*, 2004](#_ENREF_2)). This produced strains AH5246 (∆*spoVID*) and AH5367 (∆*safA*). The presence of the in-frame deletion mutations was confirmed by PCR. The sporulation phenotypes of *spoVID* and *safA* were tested using heat and lysozymere resistance tests and the spore germination phenotype was tested using the overlay method or by monitoring the drop in OD580 of a spore suspension following addition of the germinat L-Ala ([Costa *et al.*, 2006](#_ENREF_3), [Nicholson, 1990](#_ENREF_8)).

**A *cotE* insertional mutant**. A *cotE* insertional mutant was obtained by transformation of PY79 with chromosomal DNA of AH2835 ([Costa *et al.*, 2006](#_ENREF_3)) with selection for cloranfenicol resistance (Cm^R^), to produce AH5404 (Table S6).

**A *yabG* insertional mutant**. AH2850 (laboratory stock) is a derivative of the Spo^+^ MB24 strain ([Costa *et al.*, 2006](#_ENREF_3)) carrying a *yabG*::*erm* insertional allele. The *yabG*::*erm* mutation was transferred to strain PY79 by transformation with chromosomal DNA from AH2850 with selection for erythromycin resistance, yielding strain EM100 (Table S6).

**Strains harbouring *spoVID* alleles at the *amyE* locus.** The *spoVID* coding sequence, including promotor and terminator regions, was amplified with primers SpoVID-24D and SpoVID-1952R. These primers were also used in SOE-PCR to create *spoVID* versions with the deletion of region E or with variations in region E codons that result in single alanine substitutions, togheter with primers VIDDel125-136Dir and VIDDel125-136Rev for *spoVID_∆E_*, VID-L125ADir and VID-L125ARev for *spoVID_L125A_*, VID-T126ADir and VID-T126ARev for *spoVID_T126A_*, VID-I127ADir and VID-I127ARev for *spoVID_I127A_*, VID-L131ADir and VID-L131ARev for *spoVID_L131A_*, VID-I133ADir and VID-I133ARev for *spoVID_I133A_*, and VID-E134ADir and VID-E134ARev for *spoVID_E134A_*. The DNA fragments (corresponding to either *spoVID* wild-type or mutant versions) were digested with XbaI and BamHI and inserted in the same cloning sites of pMLK83 ([Karow & Piggot, 1995](#_ENREF_6)), creating pFN101-105, pFN107, pFN110 and pFN111 vectors. These plasmids were used to insert the different *spoVID* alleles at the *amyE* locus of strain AH5246 (carrying the in-frame deletion of *spoVID*, constructed as described above), creating strains AH5354 through AH5359, AH5362 and AH5363 (Table S6). These strains were used in the spore decoating and fractionation experiments, in heat and lysozyme resistance tests (Table 1) and to monitor the accumulation of the various forms of SpoVID by immunoblot (Fig. S2).

**A functional SafA-YFP fusion.** *safA* and its flanking regions were amplified using primers safA-169D and safA+1248R, digested with PstI and SalI and inserted in the same cloning sites in pUC18 (Thermo Scientific). The vector was then digested with PstI and BamHI and the fragment was inserted in the same cloning sites of pLitmus (New England Biolabs), that in turn was digested with XhoI and BamHI and the fragment harbouring *safA* and its flanking regions cloned in SalI and BamHI sites of pMLK83 (Karow and Piggot, 1994), creating pCF75 ([Fernandes *et al.*, 2018](#_ENREF_4)). A DNA fragment harboring the 3’ region of *safA*, a sequence coding for the flexible linker FL3 (residues LGGGGSGGGGSGGGGSAAA) ([Arai *et al.*, 2001](#_ENREF_1)) and the 5’ region of *yfp* was generating without template using primers safA-fl3D and fl3-yfpR. Through SOE-PCR with primers fl3-yfpD and yfp-TsafAR, this fragment was fused to *yfp*, and the resulting amplicon was used as a megaprimer to insert *fl3* and *yfp* at pCF75, resulting in pCF149, harbouring *safA-fl3-yfp* (herein *safA-yfp*). AH5367 (*safA* in*-*frame deletion mutant) was transformed with pCF149 to insert *safA-YFP* at *amyE* locus, creating AH5370. The functionality of this fusion was tested using spore heat and lysozyme resistance assays, by examining the profile of extractable coat proteins and spore germination in response to L-Ala ([Costa *et al.*, 2006](#_ENREF_3), [Nicholson, 1990](#_ENREF_8))(Fig. S1).

**Strains expressing SafA-YFP, YaaH-GFP, CotM-GFP and CotE-CFP fusions.** For SafA-YFP expression, strain AH5370, with the in-frame deletion of *safA* and harbouring *safA-yfp* inserted at the *amyE* locus was constructed as described above. To obtain a strain expressing SafA-YFP in a *spoVID* null mutant background, and in-frame deletion of *safA* was created in AH5246 (with the *spoVID* in-frame deletion) using pFN115, as previously described ([Arnaud *et al.*, 2004](#_ENREF_2)) Once the deletion was confirmed by PCR, *safA-yfp* was inserted at *amyE* locus using pCF149, creating AH5371 strain. For SafA-YFP expression in a *cotE* null mutant background, strain AH5370 was transformed with the chromosomal DNA of AH2835 for disruption of *cotE* ([Costa *et al.*, 2006](#_ENREF_3)) followed by selection for Cm^R^. The maintainance of the *safA* in-frame deletion in the resulting strain, AH5240, was confirmed by PCR. For the construction of strains expressing SafA-YFP and missing region E of SpoVID or with alanine substitutions in this region, new pMAD ([Arnaud *et al.*, 2004](#_ENREF_2)) derivatives were designed. SOE-PCR was used to amplify *spoVID* variants, using primers spoVID-17D and spoVID-1952R, so as VIDDel125-136Dir and VIDDel125-136Rev for *spoVID_∆E_*, VID-L125ADir and VID-L125ARev for *spoVID_L125A_*, VID-T126ADir and VID-T126ARev for *spoVID_T126A_*, VID-I127ADir and VID-I127ARev for *spoVID_I127A_*, VID-Q128ADir and VID-Q128ARev for *spoVID_Q128A_*, VID-D130ADir and VID-D130ARev for *spoVID_D130A_*, VID-L131ADir and VID-L131ARev for *spoVID_L131A_*, VID-I133ADir and VID-I133ARev for *spoVID_I133A_*, VID-E134ADir and VID-E134ARev for *spoVID_E134A_*, VID-G135ADir and VID-G135ARev for *spoVID_G135A_*, and VID-L136ADir and VID-L136ARev for *spoVID_L136A_*. These amplicons were digested with BglII and BamHI and inserted into the BglII site of pMAD ([Arnaud *et al.*, 2004](#_ENREF_2)) creating pFN116-126 vectors. AH5370 was transformed with these vectors as described, replacing of the wild-type *spoVID* for *spoVID* versions by double crossing-over at the locus ([Arnaud *et al.*, 2004](#_ENREF_2)). The replacement in the resulting strains, AH5383-5393, was confirmed by sequencing. To obtain cells expressing YaaH-GFP or CotM-GFP, strains AH5246, AH5354-5358 and AH5362 were transformed with chromosomal DNA of PE793 or PE787 ([McKenney & Eichenberger, 2012](#_ENREF_7)), harbouring *yaaH-gfp* and *cotM-gfp* fusions, respectively, and selected for Spec^r^. This generates the strains AH5413-5419 and AH5421-5427. For expression of SafA-YFP and CotE-CFP in the same cells, strains AH5367, AH5370, AH5371, AH5383, AH5384, AH5386, AH5389 and AH5390, with *safA-yfp*, were transformed with the chromosomal DNA of a strain harbouring *cotE-cfp* linked to a Spec^R^ marker (PE1945; McKenney and Eichenberger, unpublished), and with selection for Spec^R^. The maintenance of the *safA* and *spoVID* in-frame deletions in the new strains (AH5463 through AH5470) was comfirmed by PCR.

**Strains for the overproduction of GST-SafA and GST-SpoVID fusions.** DNA fragments corresponding to the *spoVID* coding region with the deletion of region E or with alterations in region E codons that create alanine substitutions were generated through SOE-PCR. Primers VIDBamHID and VIDFLXhoIR were used, so as VIDDel125-136Dir and VIDDel125-136Rev for *spoVID_∆E_*, VID-L125ADir and VID-L125ARev for *spoVID_L125A_*, VID-T126ADir and VID-T126ARev for *spoVID_T126A_*, VID-I127ADir and VID-I127ARev for *spoVID_I127A_*, VID-Q128ADir and VID-Q128ARev for *spoVID_Q128A_*, VID-D130ADir and VID-D130ARev for *spoVID_D130A_*, VID-L131ADir and VID-L131ARev for *spoVID_L131A_*, VID-I133ADir and VID-I133ARev for *spoVID_I133A_*, VID-E134ADir and VID-E134ARev for *spoVID_E134A_*, VID-G135ADir and VID-G135ARev for *spoVID_G135A_* and VID-L136ADir and VID-L136ARev for *spoVID_L136A_*. These amplicons were digested with BamHI and XhoI and inserted in pGEX-4T-2 in the same cloning sites to generate pFN85, pFN86 and pFN88-96 (harbouring GST fused to the N-terminal of SpoVID variants). *E. coli* CC118(DE3)/pLysS was transformed with these expression vectors, generating strains AH5290, AH5291 and AH5301-5309. For the overproduction of untagged SafA, *safA* coding region was amplified with primers SafA1364R and safA-364D and the fragment was digested with NcoI and BamHI and inserted in the same cloning sites of pACYCDuet-1 (Merck Millipore). The vector was used to transform *E. coli* BL21 (DE3), yielding strain AH5236.

**Accumulation of SpoVID variants during sporulation.** Sporulation was induced for strains PY79, AH5246, AH5354-5359, AH5362 and AH5363 by the resuspension method ([Sterlini & Mandelstam, 1969](#_ENREF_9)) and 10 ml samples were collected 2, 4 and 6 hours after resuspension. Cell were harvested by centrifugation (7500 x *g*, 10 min, at 4ºC), the pellets resuspended in French press buffer (10 mM Tris pH 8.0, 10 mM MgCI_2_, 0.5 mM EDTA, 0.2 M NaCI, 10% glicerol, 0.1 mM DTT, 1 mM phenylmethylsulphonylfluorite), and the cells were lysed in a French pressure cell, as described above. Cell extracts were quantified using the Bio-Rad mini protein system according to manufacturer’s instructions and 5 µl of SDS protein loading buffer were added to 10 µg of each sample. Proteins were resolved by SDS-PAGE 10%, transferred to nitrocellulose membranes and immunobloted using an affinity purified anti-SpoVID antibody (1:10000) ([Costa *et al.*, 2006](#_ENREF_3)). As a control for the amount of samples loaded, membranes were stripped as described and reprobed with an antibody against *E. coli* σ^70^ (Abcam) (1:1000) that recognizes *B. subtilis* σ^A^ ([Fujita & Losick, 2002](#_ENREF_5)).

**Supporting References**

Arai, R., H. Ueda, A. Kitayama, N. Kamiya & T. Nagamune, (2001) Design of the linkers which effectively separate domains of a bifunctional fusion protein. *Protein Eng* **14**: 529-532.

Arnaud, M., A. Chastanet & M. Debarbouille, (2004) New vector for efficient allelic replacement in naturally nontransformable, low-GC-content, gram-positive bacteria. *Appl Environ Microbiol* **70**: 6887-6891.

Costa, T., A.L. Isidro, C.P. Moran, Jr. & A.O. Henriques, (2006) Interaction between coat morphogenetic proteins SafA and SpoVID. *J Bacteriol* **188**: 7731-7741.

Fernandes, C.G., C.P. Moran, Jr. & A.O. Henriques, (2018) Auto-regulation of SafA assembly through recruitment of a protein cross-linking enzyme

. *J Bacteriol* **JB00066-18**: Accepted for publication.

Fujita, M. & R. Losick, (2002) An investigation into the compartmentalization of the sporulation transcription factor sigmaE in Bacillus subtilis. *Mol Microbiol* **43**: 27-38.

Karow, M.L. & P.J. Piggot, (1995) Construction of gusA transcriptional fusion vectors for Bacillus subtilis and their utilization for studies of spore formation. *Gene* **163**: 69-74.

McKenney, P.T. & P. Eichenberger, (2012) Dynamics of spore coat morphogenesis in Bacillus subtilis. *Mol Microbiol* **83**: 245-260.

Nicholson, W.L.a.S., P., (1990) Sporulation, Germination and Outgrowth. In: Molecular Biology Methods for Bacillus*.* H.C.R.a.C. S.M. (ed). Chichester: John Wiley & Sons Ltd, pp. 391-450.

Sterlini, J.M. & J. Mandelstam, (1969) Commitment to sporulation in Bacillus subtilis and its relationship to development of actinomycin resistance. *Biochem J* **113**: 29-37.

Wang, K.H., A.L. Isidro, L. Domingues, H.A. Eskandarian, P.T. McKenney, K. Drew, P. Grabowski, M.H. Chua, S.N. Barry, M. Guan, R. Bonneau, A.O. Henriques & P. Eichenberger, (2009) The coat morphogenetic protein SpoVID is necessary for spore encasement in Bacillus subtilis. *Mol Microbiol* **74**: 634-649.

Youngman, P., J.B. Perkins & R. Losick, (1984) A novel method for the rapid cloning in Escherichia coli of Bacillus subtilis chromosomal DNA adjacent to Tn917 insertions. *Mol Gen Genet* **195**: 424-433.

**Supporting Figure Legends**

**Figure S1 - Functionality of the SafA-YFP fusion**. **A:** Scheme of the new SafA-YFP construction. A linker and YFP were added to the C-terminal end of the protein. **B:** Profile of proteins extractable from the coat. The WT and strains with an in-frame deletion of *safA* (Δ*safA*), the mutant complemented with either the WT allele or the *safA*-*hl4*-*yfp* fusion at *amyE*, were grown in resuspension medium. Spores were harvested 24 hours after the onset of sporulation, purified by density gradient centifugation and the coat proteins extracted and analyzed by SDS-PAGE. The gel shown was stained with Coomassie blue. The red arrows indicate proteins that absent or reduced in the indicated strains, relative to the WT. The green arrows indicate proteins that are present in the strain expressing *safA*-*fl3*-*yfp* and absent or reduced in the deletion mutant. The position of molecluar markers (MW, in kDa) is shown in the left side of the panel. **C:** germination of WT spores (circles) or spores produced by a strain expressing *safA-yfp* in a Δ*safA* background (triangles). Spores were purified by density gradient centrifugation and heat activated. The rate and extent of germination was followed by monitoring the drop in the OD at 580 nm (OD_580_) of the suspension in the presence (closed symbols) or in the absence (opne symbols) of the germinant L-Ala. The results are expressed as the percentage of the initial OD_580_. **D**: the figure shows the titer of total (viable), heat- and lysozyme-resistant colony forming units obtained for spores of the indicated strains, grown in resuspnsion medium for 18 hours.

**Figure S2 -** SafA-YFP localization *in vivo* for all strains with single alanine substitutions in region E of SpoVID 2 (in panel A), 4 (B), and 6 (C) hours after ressuspension in SM medium. The cells were collected from sporulating cultures, stained with the membrane dye FM4-64 and examined by phase contrast and fluorescence microscopy. Scale bar, 1 µm. See also Figs. 2 and 3.

**Figure S3 - SpoVID forms with single Ala substitutions in region E accumulate in sporulating cells**. Cells were sporulated at 37ºC and samples were taken 2, 4 and 6 hours after ressuspension in SM medium. The same amount of cell extracts was loaded , resolved by SDS-PAGE and immunobloted with an anti-SpoVID antibody. Reprobing of the membranes with and anti-σ^A^ antibody served as a loading control. WT^C^*:* Δ*spoVID* strain carrying a WT *spoVID* allele inserted at the *amyE* locus.

**Figure S4 - L125, I127 and I133A are required for the localization of a SafA-dependent and a CotE-dependent protein**. **A**: strains producing the indicated GFP fusion, in either a WT background or in the indicated strains, were grown in resuspension medium and samples collected at the indicated times, in hours, after the onset of sporulation. The cells were stained with FM4-64 and examined by fluorescence microscopy. Scale bar, 1 µm. **B**: quantification of the various localization patterns depicted in cartoon form, for the indicated strains at the various sampling times. A more detailed quantification can be found in Tables S2 (YaaH-GFP) and S3 (CotM-GFP).

**Figure S5 – YaaH-GFP, larger fields of cell. A**: the strain producing a YaaH-GFP fusion in an otherwise WT background was grown and sampled as in the legend for figure S4. The figure is similar to S4 except that a larger filed of cells is shown. In the figure, the FM4-64 image is shown in black and white for improved contrast. The red arrows show sporangia during engulfment with a cap of YaaH-GFP at the MCP pole and the blue arrows show sporangia with two caps of YaaH-GFP fluorescence, following engulfment completion. Scale bar, 1 µm. **B**: quantification of the various localization patterns depicted in cartoon form, for the indicated strains at the various sampling times.

**Figure S6 – Role of YabG in the assembly of SafA.** Spores were produced in SM medium, from WT cultures or cultures of a congenic Δ*yabG* mutant, collected 18 hours after resuspension and density-gradient purified. The spores were decoated to produce a coat fraction (C); the decoated spores were then re-extracted before (“-“) or after (“+”) treatement with lysozyme ([Fernandes *et al.*, 2018](#_ENREF_14)). Proteins present on the different fractions were resolved by SDS-PAGE and immunobloted with anti-SafA, anti-CotA and anti-SpoVID antibodies. The arrows indicate the position of relevant species. In the top panel, the bands above the position of SafA^FL^ most likely represent cross-linked forms of SafA^FL^ and/or C30. Because the *yabG* mutation increases the extractability of all of the SafA forms, the spore coat extracts prepared from spores of the mutant were diluted 1:2 realtive to the amount loaded for the WT coat extracts. The position of molecular weight markers (in kDa) is show on the left side of the panels.

**Supporting Tables**

**Table S1 - Encasement by SafA-YFP in strains with various *spoVID* alleles.** The table shows the percentage of sporulating cells with the indicated patterns of localization of SafA-YFP 2, 4 and 6 hours after resuspension in SM medium, in the strains indicated on the left column.

|  | Hour 2* | | |  | Hour 4 | | | |  | Hour 6 | | | | |
| --- | --- | --- | --- | --- | --- | --- | --- | --- | --- | --- | --- | --- | --- | --- |
|  | ****** | ****** | ****** |  | ****** | ****** | ****** | ****** |  | ****** | ****** | ****** | ****** | ****** |
| WT | 3** | 91 | 6 |  | 0 | 21 | 47 | 32 |  | 0 | 10 | 7 | 83 | 0 |
| *ΔspoVID* | 84 | 16 | 0 |  | 34 | 66 | 0 | 0 |  | 6 | 59 | 0 | 0 | 35 |
| ΔE | 88 | 12 | 0 |  | 30 | 70 | 0 | 0 |  | 24 | 17 | 0 | 0 | 59 |
| L125A | 28 | 72 | 0 |  | 3 | 96 | 1 | 0 |  | 0 | 90 | 0 | 0 | 10 |
| T126A | 5 | 93 | 2 |  | 0 | 19 | 58 | 23 |  | 0 | 11 | 13 | 76 | 0 |
| I127A | 36 | 64 | 0 |  | 0 | 98 | 0 | 2 |  | 3 | 91 | 1 | 2 | 3 |
| Q128A | 4 | 89 | 7 |  | 0 | 12 | 42 | 46 |  | 0 | 11 | 7 | 80 | 2 |
| D130A | 0 | 100 | 0 |  | 0 | 28 | 33 | 39 |  | 0 | 10 | 14 | 76 | 0 |
| L131A | 1 | 95 | 4 |  | 0 | 15 | 47 | 38 |  | 0 | 13 | 16 | 71 | 0 |
| I133A | 23 | 77 | 0 |  | 0 | 60 | 16 | 28 |  | 0 | 35 | 3 | 58 | 4 |
| E134A | 6 | 94 | 0 |  | 0 | 25 | 47 | 28 |  | 0 | 17 | 13 | 70 | 0 |
| G135A | 2 | 94 | 4 |  | 0 | 28 | 27 | 45 |  | 0 | 13 | 15 | 70 | 2 |
| L136A | 3 | 96 | 1 |  | 0 | 26 | 33 | 41 |  | 0 | 15 | 11 | 74 | 0 |

***** the indicated pattern represents 4% of the sporangia in the WT at hour 2 of sporulation; it was not scored for the remaining strains (see also Fig. 2).

**The numbers refer to percentages of sporangia with the indicated patterns.

**Table S2 - Encasement by YaaH-YFP in strains with various *spoVID* alleles.** The table shows the percentage of sporulating cells with the indicated patterns of localization of YaaH-GFP 2, 4 and 6 hours after resuspension in SM medium, in the strains indicated on the left column.

|  | Hour 2 | | |  | Hour 4 | | | |  | Hour 6 | | | |
| --- | --- | --- | --- | --- | --- | --- | --- | --- | --- | --- | --- | --- | --- |
|  | ****** | ****** | ****** |  | ****** | ****** | ****** | ****** |  | ****** | ****** | ****** | ****** |
| WT | 0* | 82 | 18 |  | 0 | 17 | 47 | 36 |  | 6 | 19 | 75 | 0 |
| *ΔspoVID* | 7 | 93 | 0 |  | 0 | 100 | 0 | 0 |  | 83 | 4 | 5 | 8 |
| ΔE | 32 | 68 | 0 |  | 8 | 89 | 0 | 3 |  | 73 | 5 | 5 | 17 |
| L125A | 13 | 87 | 0 |  | 0 | 100 | 0 | 0 |  | 85 | 3 | 2 | 12 |
| I127A | 19 | 81 | 0 |  | 0 | 100 | 0 | 0 |  | 84 | 5 | 4 | 7 |
| L131A | 0 | 82 | 18 |  | 0 | 24 | 29 | 47 |  | 11 | 26 | 63 | 0 |
| I133A | 0 | 100 | 0 |  | 0 | 48 | 5 | 47 |  | 37 | 2 | 61 | 0 |

*The numbers refer to percentages of sporangia with the indicated patterns.

**Table S3 - Encasement by CotM-GFP in strains with various *spoVID* alleles.** The table shows the percentage of sporulating cells with the indicated patterns of of CotM-GFP localization 2, 4 and 6 hours after the onset of sporulation in resuspension medium, in the strains indicated on the left column.

|  | Hour 2 | | |  | Hour 4 | | |  | Hour 6 | | |
| --- | --- | --- | --- | --- | --- | --- | --- | --- | --- | --- | --- |
|  | ****** | ****** | ****** |  | ****** | ****** | ****** |  | ****** | ****** | ****** |
| WT | 55* | 41 | 4 |  | 8 | 18 | 74 |  | 0 | 10 | 90 |
| *ΔspoVID* | 44 | 56 | 0 |  | 7 | 93 | 0 |  | 0 | 100 | 0 |
| ΔE | 61 | 39 | 0 |  | 7 | 93 | 0 |  | 4 | 96 | 0 |
| L125A | 55 | 45 | 0 |  | 5 | 95 | 0 |  | 0 | 100 | 0 |
| I127A | 50 | 50 | 0 |  | 6 | 93 | 1 |  | 0 | 100 | 0 |
| L131A | 69 | 31 | 0 |  | 15 | 83 | 2 |  | 0 | 100 | 0 |
| I133A | 48 | 46 | 4 |  | 2 | 20 | 78 |  | 0 | 17 | 83 |

*****The numbers refer to percentages of sporangia with the indicated patterns.

**Table S4 - Encasement by SafA-YFP and CotE-CFP.** The table shows the percentage of sporulating cells with the indicated patterns of SafA-YFP and CotE-CFP, present in the same strain, in the strains indicated on the left column, 2, 4, 6 and 8 after the onset of sporulation in resuspension medium.

|  |  | Hour 2 | |  | Hour 4 | | | | |  | Hour 6 | | | | | | | |  | Hour 8 | | | | | | | | | |
| --- | --- | --- | --- | --- | --- | --- | --- | --- | --- | --- | --- | --- | --- | --- | --- | --- | --- | --- | --- | --- | --- | --- | --- | --- | --- | --- | --- | --- | --- |
| SafA-**YFP** |  | 1c* | |  | 1c | |  | 2c* | |  | 1c | |  | 2c | |  | S* | |  | 1c | |  | 2c | | |  | S | | |
| CotE-**CFP** |  | No | 1P |  | 1P | 2P |  | 1P | 2P |  | 1P | 2P |  | 1P | 2P |  | 1P | 2P |  | 1P | 2P |  | 1P | 2P | S |  | 1P | 2P | S |
| WT |  | 24 | **72** |  | 25 | 0 |  | 0 | **74** |  | 5 | 0 |  | 0 | **95** |  | 0 | 0 |  | 0 | 0 |  | 0 | **82** | 0 |  | 0 | 7 | 0 |
| *ΔspoVID* |  | 13 | **83** |  | **98** | 0 |  | 0 | 0 |  | **59** | 0 |  | 0 | 0 |  | 7 | 31 |  | **79** | 0 |  | 0 | 0 | 0 |  | 10 | 0 | 7 |
| ΔE |  | 10 | **88** |  | **99** | 0 |  | 0 | 0 |  | **87** | 0 |  | 0 | 0 |  | 7 | 6 |  | 27 | 0 |  | 0 | 0 | 0 |  | 16 | 0 | **53** |

*1c: one cap at the MCP spore pole; 2c: 2 caps or complete ring; S: spread in the mother cell cytoplasm. The numbers refer to percentages of sporangia with the indicated patterns.

**Table S5 - Encasement by SafA-YFP in the absence of *cotE* and encasement by CotE-CFP in the absence of *safA*.** The table shows the percentage of sporulating cells with the indicated patterns of SafA-YFP and CotE-CFP, present in the same strain, in the WT, and in Δ*cotE* or Δ*safA* deletion mutants, 2, 4, 6 and 8 of sporulation in resuspension medium.

|  | | Hour 2 |  | Hour 4 | |  | Hour 6 |  | Hour 8 |
| --- | --- | --- | --- | --- | --- | --- | --- | --- | --- |
|  |  | 1c* |  | 1c | 2c* |  | 2c |  | 2c |
| SafA-**YFP** | WT | 74 |  | 25 | 74 |  | 88 |  | 89 |
|  | Δ*cotE* | 93 |  | 22 | 75 |  | 88 |  | 95 |
|  |  |  |  |  |  |  |  |  |  |
| CotE-**CFP** | WT | 96 |  | 25 | 74 |  | 95 |  | 82 |
|  | Δ*safA* | 92 |  | 26 | 72 |  | 88 |  | 68 |

*1c: one cap at the MCP spore pole; 2c: 2 caps or complete ring. The numbers refer to percentages

of sporangia with the indicated patterns.

**Table S6 - Bacterial strains used in this study.**

| Strain | Relevant Properties | Origin/Ref. |
| --- | --- | --- |
| ***E.coli*** |  |  |
| DH5α |  | Invitrogen |
| BL21 | (DE3) | Novagen |
| CC118 | (DE3)/pLysS | Colin Manoil |
| AH2687 | CC118(DE3)/pLysS/pTC55 Amp^r^ Cm^r^ | ([Costa *et al.*, 2006](#_ENREF_3)) |
| AH2692 | CC118(DE3)/pLysS/pOZ169 Amp^r^ Cm^r^ | “ |
| AH5291 | CC118(DE3)/pLysS/pFN86 Amp^r^ Cm^r^ | This work |
| AH5301 | CC118(DE3)/pLysS/pFN88 Amp^r^ Cm^r^ | “ |
| AH5302 | CC118(DE3)/pLysS/pFN89 Amp^r^ Cm^r^ | “ |
| AH5303 | CC118(DE3)/pLysS/pFN90 Amp^r^ Cm^r^ | “ |
| AH5304 | CC118(DE3)/pLysS/pFN91 Amp^r^ Cm^r^ | “ |
| AH5305 | CC118(DE3)/pLysS/pFN92 Amp^r^ Cm^r^ | “ |
| AH5290 | CC118(DE3)/pLysS/pFN85 Amp^r^ Cm^r^ | “ |
| AH5306 | CC118(DE3)/pLysS/pFN93 Amp^r^ Cm^r^ | “ |
| AH5307 | CC118(DE3)/pLysS/pFN94 Amp^r^ Cm^r^ | “ |
| AH5308 | CC118(DE3)/pLysS/pFN95 Amp^r^ Cm^r^ | “ |
| AH5309 | CC118(DE3)/pLysS/pFN96 Amp^r^ Cm^r^ | “ |
| AH5236 | BL21(DE3)/pFN76 Cm^r^ | “ |
| ***B. subtilis*** |  |  |
| PY79 | Prototrophic derivative of *B. subtilis* 168 | ([Youngman *et al.*, 1984](#_ENREF_11)) |
| PE655 | *safA*::*safA*-*gfp* Spec^R^ | ([Wang *et al.*, 2009](#_ENREF_10)) |
| AH5370 | *∆safA*, *amyE’::safA-yfp::’amyE* Neo^r^ | This work |
| AH5371 | *∆safA*, *amyE’::safA-yfp::’amyE*, ∆*spoVID* Neo^r^ | “ |
| AH5383 | *∆safA*, *amyE’::safA-yfp::’amyE*, *spoVID_∆E_* Neo^r^ | “ |
| AH5384 | *∆safA*, *amyE’::safA-yfp::’amyE*, *spoVID_L125A_* Neo^r^ | “ |
| AH5385 | *∆safA*, *amyE’::safA-yfp::’amyE*, *spoVID_T126A_* Neo^r^ | “ |
| AH5386 | *∆safA*, *amyE’::safA-yfp::’amyE*, *spoVID_I127A_* Neo^r^ | “ |
| AH5387 | *∆safA*, *amyE’::safA-yfp::’amyE*, *spoVID_Q128A_* Neo^r^ | “ |
| AH5388 | *∆safA*, *amyE’::safA-yfp::’amyE*, *spoVID_D130A_* Neo^r^ | “ |
| AH5389 | *∆safA*, *amyE’::safA-yfp::’amyE*, *spoVID_L131A_* Neo^r^ | “ |
| AH5390 | *∆safA*, *amyE’::safA-yfp::’amyE*, *spoVID_I133A_* Neo^r^ | “ |
| AH5391 | *∆safA*, *amyE’::safA-yfp::’amyE*, *spoVID_E134A_* Neo^r^ | “ |
| AH5392 | *∆safA*, *amyE’::safA-yfp::’amyE*, *spoVID_G135A_* Neo^r^ | “ |
| AH5393 | *∆safA*, *amyE’::safA-yfp::’amyE*, *spoVID_L136A_* Neo^r^ | “ |
| AH5246 | *∆spoVID* | “ |
| AH5354 | *∆spoVID*, *amyE’::spoVID::’amyE* Neo^r^ | “ |
| AH5355 | *∆spoVID*, *amyE’::spoVID_∆E_::’amyE* Neo^r^ | “ |
| AH5356 | *∆spoVID*, *amyE’::spoVID_L125A_::’amyE* Neo^r^ | “ |
| AH5359 | *∆spoVID*, *amyE’::spoVID_T126A_::’amyE* Neo^r^ | “ |
| AH5357 | *∆spoVID*, *amyE’::spoVID_I127A_::’amyE* Neo^r^ | “ |
| AH5358 | *∆spoVID*, *amyE’::spoVID_L131A_::’amyE* Neo^r^ | “ |
| AH5362 | *∆spoVID*, *amyE’::spoVID_I133A_::’amyE* Neo^r^ | “ |
| AH5363 | *∆spoVID*, *amyE’::spoVID_E134A_::’amyE* Neo^r^ | “ |
| AH5367 | *∆safA* | “ |
| AH5404 | *∆cotE::cat* Cm^r^ | “ |
| EM100 | *yabG::erm* Em^r^ | “ |
| AH5413 | *∆spoVID*, *yaaHΩyaaH-gfp* Spc^r^ | “ |
| AH5414 | *∆spoVID*, *amyE’::spoVID::’amyE*, *yaaHΩyaaH-gfp* Neo^r^ Spc^r^ | “ |
| AH5415 | *∆spoVID*, *amyE’::spoVID_∆E_::’amyE*, *yaaHΩyaaH-gfp* Neo^r^ Spc^r^ | “ |
| AH5416 | *∆spoVID*, *amyE’::spoVID_L125A_::’amyE*, *yaaHΩyaaH-gfp* Neo^r^ Spc^r^ | “ |
| AH5417 | *∆spoVID*, *amyE’::spoVID_I127A_::’amyE*, *yaaHΩyaaH-gfp* Neo^r^ Spc^r^ | “ |
| AH5418 | *∆spoVID*, *amyE’::spoVID_L131A_::’amyE*, *yaaHΩyaaH-gfp* Neo^r^ Spc^r^ | “ |
| AH5419 | *∆spoVID*, *amyE’::spoVID_I133A_::’amyE*, *yaaHΩyaaH-gfp* Neo^r^ Spc^r^ | “ |
| AH5421 | *∆spoVID*, *cotMΩcotM-gfp* Spc^r^ | “ |
| AH5422 | *∆spoVID*, *amyE’::spoVID::’amyE*, *cotMΩcotM-gfp* Neo^r^ Spc^r^ | “ |
| AH5423 | *∆spoVID*, *amyE’::spoVID_∆E_::’amyE*, *cotMΩcotM-gfp* Neo^r^ Spc^r^ | “ |
| AH5424 | *∆spoVID*, *amyE’::spoVID_L125A_::’amyE*, *cotMΩcotM-gfp* Neo^r^ Spc^r^ | “ |
| AH5425 | *∆spoVID*, *amyE’::spoVID_I127A_::’amyE*, *cotMΩcotM-gfp* Neo^r^ Spc^r^ | “ |
| AH5426 | *∆spoVID*, *amyE’::spoVID_L131A_::’amyE*, *cotMΩcotM-gfp* Neo^r^ Spc^r^ | “ |
| AH5427 | *∆spoVID*, *amyE’::spoVID_I133A_::’amyE*, *cotMΩcotM-gfp* Neo^r^ Spc^r^ | “ |
| AH5464 | *∆safA*, *amyE’::safA-yfp::’amyE*, *cotEΩcotE-cfp* Neo^r^ Spc^r^ | “ |
| AH5465 | *∆safA*, *amyE’::safA-yfp::’amyE*, *∆spoVID,* *cotEΩcotE-cfp* Neo^r^ Spc^r^ | “ |
| AH5463 | *∆safA*, *cotEΩcotE-cfp*, Spc^r^ | “ |
| AH5420 | *∆safA*, *amyE’::safA-yfp::’amyE*, *∆cotE::cat* Neo^r^ Cm^r^ | “ |
| AH5466 | *∆safA*, *amyE’::safA-yfp::’amyE*, *spoVID_∆E_,* *cotEΩcotE-cfp* Neo^r^ Spc^r^ | “ |
| AH5467 | *∆safA*, *amyE’::safA-yfp::’amyE*, *spoVID_L125A_,* *cotEΩcotE-cfp* Neo^r^ Spc^r^ | “ |
| AH5468 | *∆safA*, *amyE’::safA-yfp::’amyE*, *spoVID_I127A_,* *cotEΩcotE-cfp* Neo^r^ Spc^r^ | “ |
| AH5469 | *∆safA*, *amyE’::safA-yfp::’amyE*, *spoVID_L131A_,* *cotEΩcotE-cfp* Neo^r^ Spc^r^ | “ |
| AH5470 | *∆safA*, *amyE’::safA-yfp::’amyE*, *spoVID_I133A_,* *cotEΩcotE-cfp* Neo^r^ Spc^r^ | “ |

**Table S7 – Oligonucleotides used in this study.**

| Primer | Sequence (5´to 3´) | Used in^1^ |
| --- | --- | --- |
| VIDBamHID | CCGCGTGGATCCTTGCCGCAAAATCATCG | pFN86-96 |
| VIDFLXhoIR | CGGCCGCTCGAGTTACGCATGGCTATTTTTATATTGAGG | pFN86-96 |
| VIDDel125-136Dir | GACTATCAATTGACGGATTCGCGCATTTTGGACGATACGCAAGACAAAGAGCCG | pFN86, pFN102 |
| VIDDel125-136Rev | CCGGCTCTTTGTCTTGCGTATCGTCCAAAATGCGCGAATCCGTCAATTGATAGT | pFN86, pFN102 |
| VID-L125ADir | CAATTGACGGATTCGCGCATTGCAACAATTCAAGCTGATTTAGC | pFN88, pFN103 |
| VID-L125ARev | CGCTAAATCAGCTTGAATTGTTGCAATGCGCGAATCCGTCAATTG | pFN88, pFN103 |
| VID-T126ADir | GACGGATTCGCGCATTTTAGCAATTCAAGCTGATTTAGCG | pFN89, pFN107 |
| VID-T126ARev | CGCTAAATCAGCTTGAATTGCTAAAATGCGCGAATCCGTC | pFN89, pFN107 |
| VID-I127ADir | CGGATTCGCGCATTTTAACAGCTCAAGCTGATTTAGCGATCG | pFN90, pFN104 |
| VID-I127ARev | CGATCGCTAAATCAGCTTGAGCTGTTAAAATGCGCGAATCCG | pFN90, pFN104 |
| VID-Q128ADir | CGCGCATTTTAACAATTGCAGCTGATTTAGCGATCG | pFN91 |
| VID-Q128ARev | CGATCGCTAAATCGACTGCAATTGTTAAAATGCGCG | pFN91 |
| VID-D130ADir | GCATTTTAACAATTCAAGCTGCTTTAGCGATCGAAGGGC | pFN92 |
| VID-D130ARev | GCCCTTCGATCGCTAAAGCAGCTTGAATTGTTAAAATGC | pFN92 |
| VID-L131ADir | CGCGCATTTTAACAATTCAAGCTGATGCTGCGATCGAAGGGCTTTTGGACGATACGC | pFN85, pFN105 |
| VID-L131ARev | GCGTATCGTCCAAAAGCCCTTCGATCGCAGCATCAGCTTGAATTGTTAAAATGCGCG | pFN85, pFN105 |
| VID-I133ADir | CAAGCTGATTTAGCGGCCGAAGGGCTTTTGGACG | pFN93, pFN110 |
| VID-I133ARev | CGTCCAAAAGCCCTTCGGCCGCTAAATCAGCTTG | pFN93, pFN110 |
| VID-E134ADir | CAAGCTGATTTAGCGATCGCAGGGCTTTTGGACGATACG | pFN94, pFN111 |
| VID-E134ARev | CGTATCGTCCAAAAGCCCTGCGATCGCTAAATCAGCTTG | pFN94, pFN111 |
| VID-G135ADir | GCTGATTTAGCGATCGAAGCGCTTTTGGACGATACGC | pFN95 |
| VID-G135ARev | GCGTATCGTCCAAAAGCGCTTCGATCGCTAAATCAGC | pFN95 |
| VID-L136ADir | GATTTAGCGATCGAAGGGGCTTTGGACGATACGCAAGAC | pFN96 |
| VID-L136ARev | GTCTTGCGTATCGTCCAAAGCCCCTTCGATCGCTAAATC | pFN96 |
| SafA201Dir | GGGGAAAACCATGGCGAAAATCCATATCG | pFN76 |
| SafA1364R | CGTTCGGATCCATCACTCATTTTCTTC | pFN76 |
| safA-364D | GCAAGTCGACAATCGGGACAGAAATGAATCTTG | pFN115 |
| safA+135R | CCCGGGTCATACTGAGGCGATACTATTTTCATTCCAGGCATGATTAAGTC | pFN115 |
| safA+748D | CATGCCTGGAATGAAAATAGTATCGCCTCAGTATGACCCGGGTTATG | pFN115 |
| safA+1250R | GGGAATTCTAAGCGTGTCAGTTCTCTCCATTTG | pFN115 |
| safA-fl3 D | CCGTCCGGAAGAAGAAAATGAGCTGGGCGGAGGCGGATCAGGCGGAGG | pCF149 |
| fl3-yfp R | GTTCTTCTCCTTTACTTGCCGCAGCTGATCCGCCTCCGCCAG | pCF149 |
| fl3-yfp D | CTGCGGCAAGTAAAGGAGAAGAACTTTTCACTG | pCF149 |
| yfp-TsafA R | GATTTACATCGTTCCGAACGATCATTTGTATAGTTCATCCATGCCATGTG | pCF149 |
| VID-UP385Dir | CCGCATACCTTTAACCGTGCAGG | pFN87 |
| VID-294Rev | CCAAGGCTAAAGAATTCATCCTGACCTGAACCCTAATATCAGGAT | pFN87 |
| VID-1846Dir | TCAGGATGAATTCTTTAGCCTTGG | pFN87 |
| VID-2290Rev | CGGTTCTCTCGTGAAGACGGGC | pFN87 |
| spoVID-17D | GAAAACAGATCTCAGGCAGCTGAGAAAG | pFN116-126 |
| spoVID-1952R | CCACATTTTCGGATCCCTTACGGTTTACGC | pFN101-105, pFN107, pFN110-111, pFN116-126 |
| spoVID24D | CAATCTAGACAGCTGAGAAAG | pFN101-105,  pFN107, pFN110-111 |

^1^used in the construction of the indicated plasmids; see the Supporting Material and Methods for a detailed description of all plasmid constructions.

**Table S8 – Plasmids used in this study.**

| Plasmid^1^ | Relevant features | Origin/References |
| --- | --- | --- |
| pTC55 | pGEX-4T3 derivative for GST overexpression | ([Costa *et al.*, 2006](#_ENREF_3)) |
| pOZ169 | pGEX-4T3 derivative for GST-SpoVID overexpression | “ |
| pFN86 | pGEX-4T2 derivative for GST-SpoVID_∆E_ overexpression | “ |
| pFN88 | pGEX-4T2 derivative for GST-SpoVID_L125A_ overexpression | “ |
| pFN89 | pGEX-4T2 derivative for GST-SpoVID_T126A_ overexpression | “ |
| pFN90 | pGEX-4T2 derivative for GST-SpoVID_I127A_ overexpression | “ |
| pFN91 | pGEX-4T2 derivative for GST-SpoVID_Q128A_ overexpression | “ |
| pFN92 | pGEX-4T2 derivative for GST-SpoVID_D130A_ overexpression | “ |
| pFN85 | pGEX-4T2 derivative for GST-SpoVID_L131A_ overexpression | “ |
| pFN93 | pGEX-4T2 derivative for GST-SpoVID_I133A_ overexpression | “ |
| pFN94 | pGEX-4T2 derivative for GST-SpoVID_E134A_ overexpression | “ |
| pFN95 | pGEX-4T2 derivative for GST-SpoVID_G135A_ overexpression | “ |
| pFN96 | pGEX-4T2 derivative for GST-SpoVID_L136A_ overexpression | “ |
| pFN76 | pACYCDuet-1 derivative for SafA overexpression | “ |
| pFN115 | pMAD derivative to perform an *in frame* deletion of *safA* | “ |
| pCF149 | pMLK83 derivative for insertion of *safA-yfp* at *amyE* locus | “ |
| pFN87 | pMAD derivative to perform an *in frame* deletion of *spoVID* | “ |
| pFN116 | pMAD derivative for deletion of region E in *spoVID* | “ |
| pFN117 | pMAD derivative for substitution L125A in *spoVID* | “ |
| pFN118 | pMAD derivative for substitution T126A in *spoVID* | “ |
| pFN119 | pMAD derivative for substitution I127A in *spoVID* | “ |
| pFN120 | pMAD derivative for substitution Q128A in *spoVID* | “ |
| pFN121 | pMAD derivative for substitution D130A in *spoVID* | “ |
| pFN122 | pMAD derivative for substitution L131A in *spoVID* | “ |
| pFN123 | pMAD derivative for substitution I133A in *spoVID* | “ |
| pFN124 | pMAD derivative for substitution E134A in *spoVID* | “ |
| pFN125 | pMAD derivative for substitution G135A in *spoVID* | “ |
| pFN126 | pMAD derivative for substitution L136A in *spoVID* | “ |
| pFN101 | pMLK83 derivative for insertion of *spoVID* at *amyE* locus | “ |
| pFN102 | pMLK83 derivative for insertion of *spoVID_∆E_* at *amyE* locus | “ |
| pFN103 | pMLK83 derivative for insertion of *spoVID_L125A_* at *amyE* locus | “ |
| pFN107 | pMLK83 derivative for insertion of *spoVID_T126A_* at *amyE* locus | “ |
| pFN104 | pMLK83 derivative for insertion of *spoVID_I127A_* at *amyE* locus | “ |
| pFN105 | pMLK83 derivative for insertion of *spoVID_L131A_* at *amyE* locus | “ |
| pFN110 | pMLK83 derivative for insertion of *spoVID_I133A_* at *amyE* locus | “ |
| pFN111 | pMLK83 derivative for insertion of *spoVID_E134A_* at *amyE* locus | “ |

^1^see the Supporting Material and Methods for a detailed description of all plasmid constructions.
